# Synthetic avenues towards a tetrasaccharide related to *Streptococcus pneumonia* of serotype 6A

**DOI:** 10.3762/bjoc.14.95

**Published:** 2018-05-17

**Authors:** Aritra Chaudhury, Mana Mohan Mukherjee, Rina Ghosh

**Affiliations:** 1 Department of Chemistry, Jadavpur University, 188, Raja S. C. Mullick Rd., Kolkata 700032, India; 2 Department of Chemical Sciences, Indian Institute of Science Education and Research, Kolkata, Mohanpur, 741246, West Bengal, India; 3 present addrress: Laboratory of Bioorganic Chemistry, NIH, NIDDK, Bethesda, MD, USA

**Keywords:** carbohydrates, glycosylation, oligosaccharides, stereoselectivity, total synthesis

## Abstract

*Streptococcus pneumonia* (SPn) is a Gram-positive bacterium which causes life threatening diseases. The bacteria protect themselves against non-specific host defence by an external polysaccharide (PS) capsule which bears a repeating unit, α-D-Galp(1->3)-α-D-Glcp(1->3)-α-L-Rhap(1->3)-D-Rib (SPn 6A). A closer look at the structure reveals the presence of α-linked galactose and glucose residues. The synthesis of these 1,2-*cis* glycosidic linkages are considered challenging particularly in the context of a one-pot oligosaccharide synthesis. We have synthesized the aforesaid tetrasaccharide (SPn 6A) based on both stepwise and sequential one-pot glycosylation reactions using easily accessible common building blocks; eventually similar overall yields were obtained in both cases.

## Introduction

**Figure 1:**
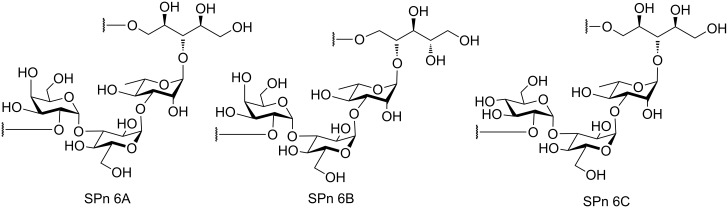
The tetrasaccharides associated with the pneumonicoccal serogroup 6.

Complex glycans serve as attractive targets for carbohydrate-based vaccines and therapeutics [1–3]. *Streptococcus pneumonia* (SPn) has been posing a serious threat in recent times. It is a major cause of pneumonia, bacteraemia, and meningitis in immune-compromised patients, elderly and children. A UNICEF/WHO survey has estimated that 920136 children died of pneumonia in 2015 accounting for 16% of all fatalities under the age of five [4]. Out of over 90 serotypes that have been reported for SPn [5–6], serogroup 6 has been ranked among the most important causes of invasive pneumonococcal diseases [7]. These facts have led to extensive research towards the establishment of polysaccharide structures associated with the SPn serogroup 6 [8–9] (Figure 1).

Initially, it was thought that due to their similar carbohydrate core structures the antibodies elicited by SPn 6A would be effective against SPn 6B as well [10–13]. But recent studies have shown that serotype specific immune responses are elicited by the antibodies and that they cross react slowly [14]. As a result the importance of the presence of the capsular polysaccharide of SPn 6A in multicomponent vaccines like Pneumovax^®^ has been recognized [15]. The low hydrolytic stability of the phosphodiester linkages in the clinical isolates of the SPn 6A polysaccharides poses a major drawback as it leads to low bioavailability [16]. Hence, the requirements of pure SPn 6A conjugate in proper amounts for future vaccine development can only be met via chemical synthesis. Therefore, a number of syntheses targeting the SPn 6A tetrasaccharide has been reported in literature. Initial reports of a linear synthesis were made by Vliegenthart et al. in the nineties [17–21]. After this, the Demchenko group improved these early reports with a convergent approach using glycosyl thioimidates as complementary glycosyl donors with respect to thioglycosides [22–25]. Herein, we wish to report synthetic routes to the SPn 6A tetrasaccharide via stepwise as well as one-pot sequential glycosylation strategies.

## Results and Discussion

**Figure 2:**
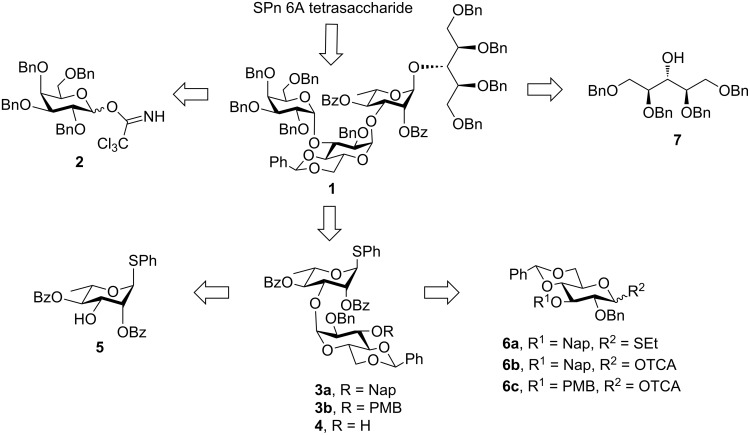
Retrosynthetic analysis.

Keeping in mind our objective to synthesize the SPn 6A tetrasaccharide following stepwise as well as one-pot synthetic strategies based on common building blocks, a retrosynthetic analysis was made which led us to galactose-based donor **2** [26], ribitol-based acceptor **7** [22] and a gluco-rhamno-based disaccharide (**3a**/**3b**) to contemplate the synthesis of the tetrasaccharide derivative **1** (Figure 2).

The disaccharides (**3a**/**3b**) can be synthesized from their parent monomeric units **6a/6b/6c** and **5** [27]. To ensure high α-selection during the formation of the central gluco-rhamno disaccharide the benzylidene protected glucosyl donor (Figure 2) was selected, because the induction of the 1,2-*cis* selectivity in benzylidene-protected substrates via torsional/electronic effects have already been recognized [28].

**Scheme 1:**
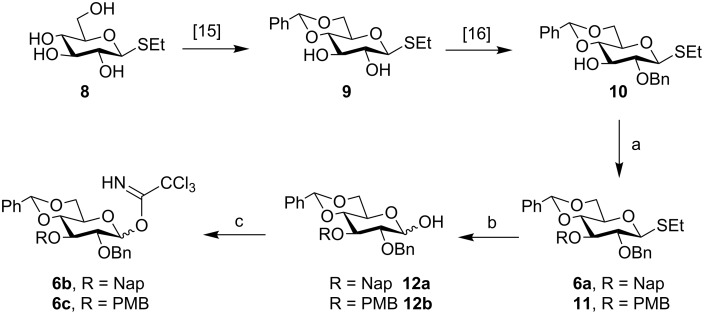
Preparation of D-glucosyl donor **6**. Reaction conditions: a) NapBr/PMBCl, NaH, DMF, rt, 12 h, 90% (**6a**), 80% (**11**); b) TCCA, CH_3_COCH_3_/H_2_O (4:1), rt, 85% (**12a**), 89% (**12b**); c) CCl_3_CN, DBU, DCM, 0 °C, 93% (**6b**), 90% (**6c**).

The galactosyl trichloroacetimidate donor **2** was prepared following literature procedures [26]. On the other hand the D-glucosyl thioglycoside **8** was converted to the known benzylidene derivative **9** [29–30] according to our previously reported procedure. Benzylation of **9** under phase-transfer conditions led to **10** [31] in 49% yield (Scheme 1). Subsequently, **10** was subjected to naphthylmethylation/*p*-methoxybenzylation [32] with 2-(bromomethyl)naphthalene (NapBr)/*p*-methoxybenzyl chloride in DMF to afford **6a**/**11** [32] in 90% and 80% yields, respectively.

These derivatives were next subjected to thioglycoside hydrolysis using trichloroisocyanuric acid (TCCA) [33] in wet acetone which provided **12a/12b** [34] in 85% and 89% yields, respectively. These were finally converted to their corresponding trichloroacetimidate **6b**/**6c** [34] (Scheme 1) with yields of 93% and 90%, respectively.

**Scheme 2:**
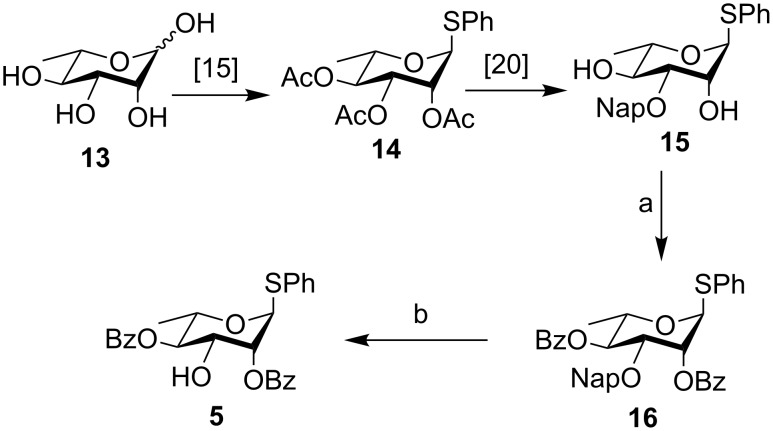
Preparation of L-rhamnosyl acceptor **5**. Reaction conditions: a) Py, BzCl, rt, 12 h, 99%; b) DDQ, DCM/H_2_O (19:1), rt, 2 h, 83%.

The L-rhamnosyl thioglycoside **14** [29–30], prepared from L-rhamnose (**13**), was deacetylated quantitatively in the presence of Et_3_N/MeOH/H_2_O [35], and then stannylene-mediated selective naphthylmethylation at the O-3 position was carried out to give the known derivative **15** in 82% yield [36]. This was next benzoylated almost quantitatively to give **16**. Finally DDQ-mediated deprotection of the naphthylmethyl group gave the acceptor **5** [27] in 83% yield (Scheme 2).

**Scheme 3:**
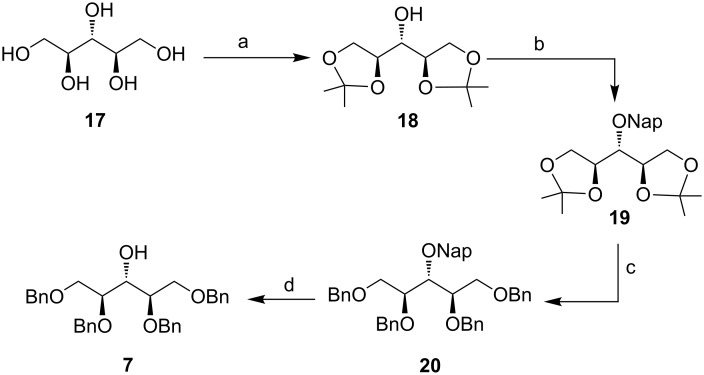
Preparation of ribitol acceptor **7**. Reaction conditions: a) Me_2_C(OMe)_2_, *p*TSA, CH_3_COCH_3_, rt, 81%; b) NapBr, NaH, DMF, rt, 8 h, 93%; c) (i) *p*TSA, MeOH, 40 °C, 4 h, (ii) BnBr, NaH, DMF, rt, 12 h, 95% over 2 steps; d) DDQ, DCM/H_2_O (19:1), rt, 2 h, 85%.

For acceptor **7** (Figure 2) ribitol **17** was converted to its corresponding diisopropylidene derivative **18** [37] in the presence of dimethoxypropane and *p*TSA in acetone. Treatment with NapBr and NaH in DMF gave compound **19** in 93% yield. Subsequent deprotection of isopropylidene ketal with *p*TSA/MeOH (aq) and then benzylation furnished **20** in 95% yield over two steps. Deprotection of the naphthylmethyl group in the presence of DDQ in aqueous dichloromethane (19:1) gave the glycosyl acceptor **7** [22] in 85% yield (Scheme 3).

**Scheme 4:**
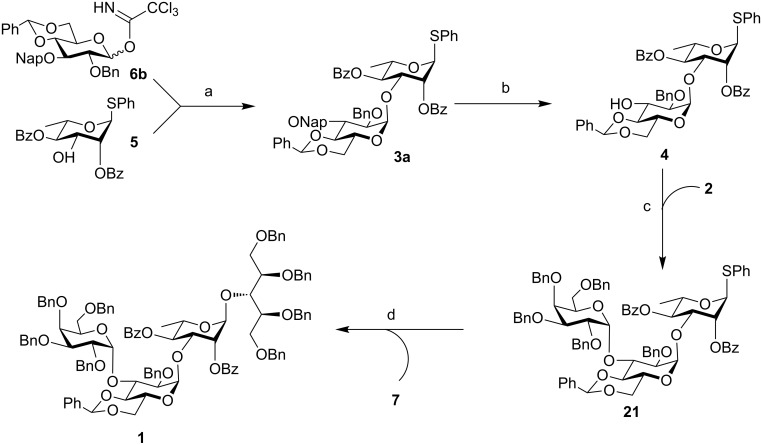
Stepwise synthesis of tetrasaccharide **1**. Reaction conditions: a) TMSOTf, DCM/Et_2_O (5:1), 4 Å MS, −30 °C, 75%; b) DDQ, DCM/H_2_O (9:1), rt, 93%; c) TMSOTf, DCM/Et_2_O (4:1), 4 Å MS, −15 °C, 70%; d) NIS, TMSOTf, DCM, 4 Å MS, −20 °C, (procedure D, see Supporting Information File 1), 89%.

In order to construct the central disaccharide fragment **3** in high yield with 1,2-*cis* selectivity, several glycosylation reactions using glucosyl donors **6a/6b/6c/12a** and rhamnosyl acceptor **5** were contemplated. None of the conditions, based on the use of thioglycoside **6a** as the glycosyl donor and separately, BSP/Tf_2_O (Table 1, entry 1) or Ph_2_SO/Tf_2_O (Table 1, entry 2) as the corresponding activators, or based on 1-hydroxy donor **12a** and Ph_2_SO/Tf_2_O (Table 1, entry 3), could furnish any desired result. After trying with the mentioned donors, and reagent combinations (Table 1), we switched over to utilize trichloroacetimidate donors (**6b**, Table 1, entries 5 and 6, and **6c**, entry 4); the TMSOTf mediated glycosylation in DCM/Et_2_O solvent (Table 1, entry 6) was found to be effective in case of donor **6b** and acceptor **5** which generated the desired disaccharide **3a** in high yield and exclusive α-anomeric selectivity (evidenced from NMR). We presume that this near exclusivity in α-selection may be due to the synergistic effect from the 4,6-O-benzylidene group, which is a good promoter for 1,2-*cis* glycosylation in galactose-based systems [38], as well as the steric crowding caused by the bulky 3-*O*-naphthylmethyl group at the β-side of the ring. Having obtained the central disaccharide **3a** in requisite yield and excellent stereochemical purity we now proceeded towards the synthesis of the trisaccharide fragment **21** (Scheme 4).

**Table 1:** Optimization of protocol for the synthesis of disaccharide **3**.

Entry	Donor	Acceptor	Conditions	Yield^a^

1	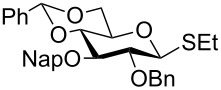 **6a**	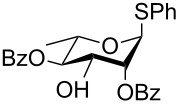 **5**	BSP^b^, Tf_2_O, DCM, −60 °C→rt (A)^c^	N.R.^d^
2	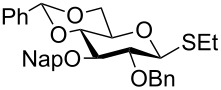 **6a**	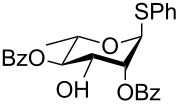 **5**	Ph_2_SO, TTBP, Tf_2_O, DCM, −60 °C→−40 °C (B)^c^	N.R.^e^
3	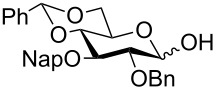 **12a**	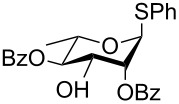 **5**	Ph_2_SO, TTBP, Tf_2_O, DCM, −60 °C→−40 °C (B)^c^	N.R.^d^
4	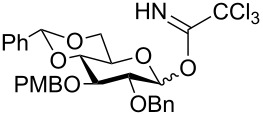 **6c**	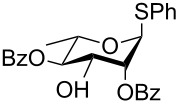 **5**	TMSOTf, DCM, −30 °C (C)^c^	N.R.^f^
5	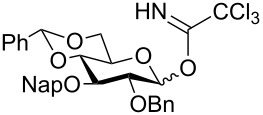 **6b**	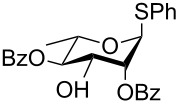 **5**	TMSOTf, DCM, −10 °C (C)^c^	**3a,** 54% (α only)
6	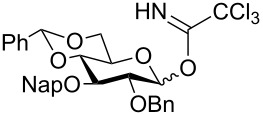 **6b**	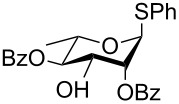 **5**	TMSOTf, DCM/Et_2_O (5:1), −30 °C (C)^c^	**3a,** 75% (α only)

^a^Isolated yields of products; ^b^BSP = benzenesulfinylpiperidine; ^c^corresponding glycosylation procedure (see Supporting Information File 1); ^d^starting material was decomposed; ^e^donor was decomposed but acceptor was recovered; ^f^a complex mixture was formed from which the desired disaccharide could not be purified by column chromatography.

Compound **3a** was treated with DDQ in dichloromethane to remove the 3-*O*-Nap protection group generating acceptor **4** in 93% yield. Glycosylation between donor **2** and acceptor **4** was achieved uneventfully in the presence of TMSOTf in dichloromethane/Et_2_O (4:1) to give the trisaccharide **21** in 70% yield. Successful glycosylation was also carried out between trisaccharide **21** and ribitol acceptor **7** in the presence of NIS and TMSOTf in dichloromethane at −20 °C to give the tetrasaccharide derivative **1** in 89% yield, thereby finishing the stepwise synthesis of SPn 6A tetrasaccharide **1** in the protected form (Scheme 4).

Having standardized a stepwise synthesis of the tetrasaccharide **1** in the protected form we then turned our attention to devise a one-pot protocol to achieve the same derivative. The one-pot synthesis of this target is particularly challenging because of the presence of the two 1,2-*cis* glycosidic linkages which are likely to make product isolation particularly difficult at the end of the glycosylative protocol. Assuming equal preference of formation for each and every possible diastereomer across the three glycosylation steps a mixture of 8 different diastereomers may be formed if the four monomers are sequentially added in a (1 + 1 + 1 + 1) one-pot strategy. However, the number of possibilities may be reduced to 4 isomers by using a participating group on the rhamnose residue to induce near exclusive α-selectivity in the last step. Further reduction can be ensured by incorporating a (1 + 2 + 1) approach where the number of possible diastereomers becomes 2. So, we selected this (1 + 2 + 1) glycosylation for our synthesis.

**Scheme 5:**
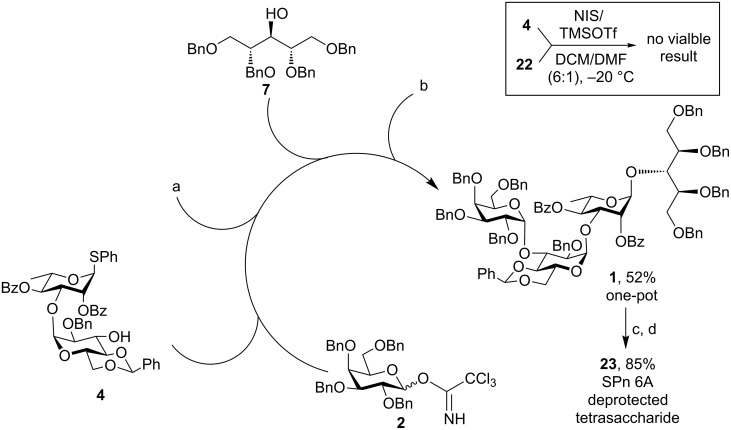
One-pot synthesis of tetrasaccharide **1**. Reaction conditions: a) TMSOTf, 4 Å MS, DCM/Et_2_O (4:1), N_2_, −15 °C, 1 h; b) NIS, TMSOTf, −10 °C, 45 min; c) NaOMe, MeOH, rt; d) H_2_, Pd/C, EtOH/EtOAc/AcOH, rt.

Two different strategies were attempted in this direction. Recently, Mong et al. have reported high α-selectivity in the formation of glucan and galactan under non-participating conditions from the O-2 protecting group [39–40]. With this method, we tried to couple donor *p*-tolyl 2,3,4,6-tetra-*O*-benzyl-1-thio-β-D-galactopyranoside (**22**) with acceptor **4** using a NIS/TMSOTf combination in the presence of DMF acting as a modulating solvent (inset, Scheme 5). Unfortunately, when this strategy was applied to our case it could not produce a viable result. So we switch to the conventional orthogonal strategy for a one-pot synthesis of the targeted tetrasaccharide (Scheme 5).

The disaccharide acceptor **4** was glycosylated with galactosyl trichloroacetimidate donor **2** at −15 °C using 30 mol % of TMSOTf. After full consumption of the starting materials (TLC), into the same pot the second acceptor **7** followed by NIS were added. The reaction mixture was allowed to reach −10 °C before another 30 mol % of TMSOTf were added; TLC after 45 minutes showed complete consumption of the starting materials. Thus the targeted tetrasaccharide derivative was prepared via a three component, one-pot sequential glycosylation technique in 52% yield (Scheme 5). It is to be noted that the temperature had to be raised to −10 °C from −20 °C in the second step of the one-pot protocol. This was necessary to improve the overall yield of the final product. The tetrasaccharide derivative **1** was next deprotected under Zemplén conditions [41], followed by hydrogenation with H_2_/Pd-C in EtOH/EtOAc/AcOH solvent to give the deprotected tetrasaccharide **23** in 85% yield over two steps.

^1^H NMR in D_2_O of the target tetrasaccharide **23** showed the anomeric protons of the galactose, glucose, and rhamnose residues from the non-reducing end appearing at δ 5.32 (d, *J =* 3.5 Hz), δ 5.02 (d, *J* = 3 Hz), and δ 4.93 (bs), respectively. ^13^C NMR along with the HSQC in the same solvent revealed that the chemical shifts of the anomeric carbons of the same units from the non-reducing end are at δ 99.2 (^1^*J*_C1-H1_ = 167.7 Hz), 95.4 (^1^*J*_C1-H1_ = 169.3 Hz) and 100.1 (^1^*J*_C1-H1_ = 169.2 Hz), respectively. The values are indicative of α-stereochemistry at all the anomeric centers [42]. Moreover, the chemical shifts were found to be in fair agreement with the reported C-1 chemical shifts at δ 99.5, 95.6, and 100.3, exhibited in D_2_O corresponding to the anomeric centers of compound **23** [22]*.*

## Conclusion

Summarizing our work we have achieved stepwise and sequential one-pot syntheses of the tetrasaccharide repeating unit of SPn 6A via an orthogonal glycosylation strategy using commonly used trichloroacetimidate and thioglycoside donors. The challenging 1,2-*cis* linkages could be prepared with a yield and a selectivity which were high enough to allow the one-pot synthesis.

## Supporting Information

File 1 Experimental details for the preparation of compounds **1**, **3a**, **4**, **5**, **6a**, **6b**, **7**, **12a**, **19**, **20**, **21**, and **23** and the corresponding characterization data.

File 2 ^1^H and ^13^C NMR of compounds **1**, **3a**, **4**, **5**, **6a**, **6b**, **7**, **12a**, **19**, **20**, **21**, and **23** and 2D NMR (COSY, HSQC and HMBC) of compound **23**.
