# Supplementary material for: Synthetic avenues towards a tetrasaccharide related to Streptococcus pneumonia of serotype 6A
Source: Beilstein J Org Chem. 2018 May 17;14:1095–102. doi: 10.3762/bjoc.14.95 (PMC6009338; doi:10.3762/bjoc.14.95)
Supplement: File 1 — Experimental details for the preparation of compounds 1, 3a, 4, 5, 6a, 6b, 7, 12a, 19, 20, 21, and 23 and the corresponding characterization data. [file Beilstein_J_Org_Chem-14-1095-s001.pdf]

Supporting Information File 1
for
Synthetic avenues towards a tetrasaccharide related to *Streptococcus pneumonia* of serotype 6A

Aritra Chaudhury[1,2], Mana Mohan Mukherjee[1,3] and Rina Ghosh[1*]

Address:Department of Chemistry, Jadavpur University, 188, Raja S. C. Mullick Rd., Kolkata 700032, India, [2]present address: Department of Chemical Sciences, Indian Institute of Science Education and Research, Kolkata, Mohanpur, 741246, West Bengal, India and [3]present addrress: Laboratory of Bioorganic Chemistry, NIH, NIDDK, Bethesda, MD, USA

Email: Rina Ghosh[*]- ghoshrina@yahoo.com

* Corresponding author

Experimental details for the preparation of compounds 1, 3a, 4, 5, 6a, 6b, 7, 12a, 19, 20, 21, and 23 and the corresponding characterization data

Experimental

General remarks	S2
Preparation of compound **6a**	S3
Preparation of compound **12a**	S4
Preparation of compound **6b**	S5
Preparation of compound **5**	S6
Preparation of compounds **19** and **20**	S7–S8
Preparation of compound **7**	S9
General procedures for glycosylation	S10–S11

Preparation of compound **3a**	S12
Preparation of compound **4**	S12
Preparation of compound **21**	S14
Preparation of compound **1**	S15
One pot preparation of compound **1**	S16
Preparation of compound **23**	S16–S17
References	S17

Experimental

General remarks

For reactions under anhydrous conditions, all glassware was stored in the oven and was flame-dried prior to use. All reagents and solvents were commercially available. Reagents were used without further purification, and solvents were distilled prior to use. Dry dichloromethane (DCM) was obtained by distillation over P_2O_5. Dimethylformamide (DMF) was distilled over calcium hydride, and methanol (MeOH) was dried over magnesium turnings. Thin layer chromatography (TLC) was done on glass plates coated with silica gel or on Merck silica gel plates (60-F_{254}) to monitor the reactions. Elution was carried out with ethyl acetate/petroleum ether (EA/PE) unless specified otherwise. Visualization of spots was accomplished by spraying the chromatograms with 5% ethanolic solution of sulfuric acid followed by charring on a hot-plate. Until otherwise stated, column chromatography was performed using 60–120 and flash column chromatography with 230–400 mesh silica, petroleum ether (PE, 60–80 °C) was used for chromatographic purpose. NMR spectra were recorded on NMR spectrometers operating at 300 MHz and 500 MHz for ^{1}H NMR and at 75 MHz and 125 MHz for ^{13}C NMR in $CDCl_3$/D_2O. Peak assignments were obtained using ^{1}H-^{1}H COSY, and ^{1}H-^{13}C

HSQC and HMBC experiments for compound **23**. Mass spectral data were recorded by HRMS (ESI-TOF). Specific rotations were measured on a JASCO (P-1020) digital polarimeter using a cell of 50 mm-path length.

Ethyl 2-*O*-benzyl-4,6-*O*-benzylidene-3-*O*-naphth-2-ylmethyl-1-thio-β-D-glucopyranoside (6a)

Ethyl 2-*O*-benzyl-4,6-*O*-benzylidene-1-thio-β-D-glucopyranoside **10** (449 mg, 1.12 mmol) was dissolved in DMF (15 mL). To the reaction mixture 2-(bromomethyl) naphthalene (0.494 mg, 2.24 mmol) was added, and the temperature was brought down to 0 °C. Then, NaH (90 mg, 2.24 mmol) was added in parts. After completion of the addition the reaction mixture was stirred at room temperature for 12 h. The reaction mixture was quenched with MeOH. The DMF was removed under reduced pressure and the crude residue was dissolved in CH_2Cl_2 and washed with brine solution (100 mL × 1). The aqueous layer was extracted with CH_2Cl_2 (40 mL × 4). The combined organic layers were dried over anhydrous Na_2SO_4 and the CH_2Cl_2 was removed under reduced pressure. The residue so obtained was purified by column chromatography (5% EA/PE) to give the product **6a** (547 mg, 90%) as white foam.

$[\alpha]_D^{25.9}$= -18.18 (c=4.04, $CHCl_3$).

^{1}H NMR (300 MHz, $CDCl_3$): δ 1.36 (3H, t , *J*= 7.4 Hz, C*H*$_3$), 2.76-2.85 (2H, m, C*H*$_2$), 3.50-3.57 (2H, m), 3.80-3.91 (3H, m), 4.40 (1H, m, *H*-6), 4.62 (1H, d, *J*= 9.7 Hz, *H*-1), 4.86-5.16 (4H, m, Nap &Bn-*H*), 5.64 (1H, s, PhC*H*), 7.34-7.36 (2H, m, Ar*H*), 7.41-7.72 (5H, m, Ar*H*), 7.47-7.52 (5H, m, Ar*H*), 7.71 (1H, m, Ar*H*), 7.77-7.85 (3H, bs, Ar*H*).

^{13}C NMR (75 MHz, $CDCl_3$): δ 15.1 (CH_3), 25.2(CH_2), 68.8, 70.3, 75.2, 75.9, 81.4, 81.6, 82.7, 85.9 (*C*-1), 101.2 (Ph*C*H), 125.8, 125.9, 126.1, 126.2, 126.8, 127.7, 127.9, 127.9, 128.1, 128.3, 128.4, 128.5, 129.0, 133.0, 133.3, 135.9, 137.3, 138.0.

HRMS: (ESI-TOF, m/z) calcd for $C_{33}H_{34}O_5SNa$ 565.2025 found 565.2024.

2-*O*-Benzyl-4,6-*O*-benzylidene-3-*O*-naphth-2-ylmethyl-D-glucopyranose (12a)

Ethyl 2-*O*-benzyl-4,6-*O*-benzylidene-3-*O*-naphth-2-ylmethyl-1-thio-β-D-glucopyranoside (**6a**, 0.94 g, 1.74 mmol) was dissolved in 4:1 acetone/water (30 mL). The reaction mixture was placed in an ice-bath and TCCA (0.4 g, 1.74 mmol) was added. The reaction mixture was brought up to room temperature and allowed to stir for 45 min. TLC at this point showed the reaction to be complete. The solvent was removed under reduced pressure and the crude residue was dissolved in CH_2Cl_2 and washed with $NaHCO_3$ solution (50 mL × 1). The aqueous layer was extracted with CH_2Cl_2 (20 mL × 4). The combined organic layers were dried over anhydrous Na_2SO_4 and the CH_2Cl_2 was removed under reduced pressure. The crude residue was purified by column chromatography (15% EA/PE) to give the product **12a** (733 mg, 85%) as a syrupy anomeric mixture.

^{1}H NMR (300 MHz, $CDCl_3$): δ 3.44-3.47 (2H, m), 3.60-3.90 (6H, m), 4.08-4.11 (3H, m), 4.30-4.39 (2H, m), 4.74 (1H, m), 4.81-5.04 (5H, m), 5.07-5.22 (3H, m), 5.29-5.33 (1H, m), 5.59 (2H, s), 7.27-7.82 (30H, m), 8.27-8.33 (1H, m).

^{13}C NMR (75 MHz, $CDCl_3$): δ 62.5, 66.2, 68.7, 69.1, 71.2, 73.8, 75.1, 75.3, 78.3, 79.5, 79.9, 80.8, 81.5, 82.0, 83.2, 92.2, 97..8, 101.3, 101.4, 125.9, 126.0, 126.1, 126.2, 126.2,

126.7, 126.8, 127.7, 127.8, 128.0, 128.1, 128.3, 128.5, 128.6, 129.1, 129.8, 133.0, 137.7.

HRMS: (ESI-TOF, m/z) calcd for $C_{31}H_{30}O_6Na$ 521.1940 found 521.1939.

2-*O*-Benzyl-4,6-*O*-benzylidene-3-*O*-naphth-2-ylmethyl-D-glucopyranosyl trichloroacetimidate (6b)

2-*O*-Benzyl-4,6-*O*-benzylidene-3-*O*-naphth-2-ylmethyl-D-glucopyranose **12a** (227 mg, 0.455 mmol) was dissolved in CH_2Cl_2 (4 mL) and CCl_3CN (0.1 mL, 0.91 mmol) was added. DBU (20 μL, 0.137 mmol) was added and the reaction mixture was allowed to stir at 0 °C for 4 h. After the reaction was complete the reaction mixture was quickly purified by elution chromatography (8% EA/PE) to give a syrupy product **6b** (270 mg, 93%). Due to its unstable nature this compound was not characterized and was carried forward to the subsequent glycosylation step as soon as it was prepared.

Phenyl 2,4-di-*O*-benzoyl-3-*O*-naphth-2-ylmethyl-1-thio-α-L-rhamnopyranoside (16)

Phenyl 3-*O*-naphth-2-ylmethyl-1-thio-α-L- rhamnopyranoside(**15** [1], 590 mg, 1.52 mmol) was dissolved in pyridine (8 mL) and then the temperature was lowered to 0 °C. Benzoyl chloride (0.52 mL, 4.5 mmol) was added. The reaction mixture was allowed to stir at room temperature overnight. After the completion of the reaction the solvent was removed under reduced pressure. The residue was dissolved in CH_2Cl_2 and washed with water (100 mL × 1). The aqueous layers were extracted with CH_2Cl_2 (20 mL × 4).The combined organic layer was dried over anhydrous Na_2SO_4. The CH_2Cl_2 was next

removed under reduced pressure. The crude product was purified by column chromatography (5% EA/PE) to give the product **16** (880 mg, 99%) as a white foam.

$[\alpha]_D^{25.4}$= +1.78 (c=1.78, $CHCl_3$).

^{1}H NMR (500 MHz, $CDCl_3$): δ 1.34 (3H, d, *J*= 6.5 Hz), 4.11 (1H, dd, *J*= 3.5 Hz, 10.0 Hz, *H*-3), 4.45 (1H, m, *H*-5), 4.68 (1H, d, *J*= 12.5 Hz, Nap-*H*), 4.85 (1H, d, *J*= 12.5 Hz, Nap-*H*), 5.57 (1H, t, *J*= 9.5 Hz, *H*-4), 5.7 (1H, d, *J*= 1.0 Hz, *H*-1), 5.97 (1H, app t, *J*= 2.0, 2.5 Hz, *H*-2), 7.24-7.34 (4H, m), 7.40-7.65 (14H, m, Ar*H*), 7.75 (1H, m, Ar*H*), 8.00 (1H, d, *J*= 7.5 Hz, Ar*H*), 8.16 (1H, d, *J*= 7.5 Hz, Ar*H*).

^{13}C NMR (125 MHz, $CDCl_3$): δ 17.7 (C*H_3*), 68.3, 70.7, 71.2, 73.3, 74.7, 86.3 (C-1), 125.9, 126.1, 127.0, 127.7, 127.9, 128.0, 128.3, 128.5, 128.6, 129.3, 129.7, 129.9, 130.0, 130.1, 131.9, 133.1, 133.2, 133.3, 133.5, 133.6, 134.9, 165.8, 165.9 (C=O).

HRMS: (ESI-TOF, m/z) calcd for $C_{37}H_{32}O_7SNa$ 627.1817 found 627.1815.

Phenyl 2,4- di-*O*-benzoyl-1-thio-α-L-rhamnopyranoside (5) [2]

Phenyl 2,4-di-*O*-benzoyl-3-*O*-naphthylmethyl-1-thio-α-L-rhamnopyranoside (**16**, 830 mg, 1.37 mmol) was dissolved in 19:1 CH_2Cl_2/H_2O (10 mL) and DDQ (360 mg, 1.58 mmol) was added. The reaction mixture was stirred at room temperature for 2 h. TLC analysis at this point showed the reaction to be complete. The reaction mixture was diluted with CH_2Cl_2 (20 mL) and washed with $NaHCO_3$ (aq) (100 mL × 1). The aqueous layer was extracted with CH_2Cl_2 (25 mL × 4).The combined organic layer was dried over anhydrous Na_2SO_4. The CH_2Cl_2 was next removed under reduced pressure. The crude product was purified by flash column chromatography (18% EA/PE) to give the product **5** (530 mg, 83%) white foam.

$[\alpha]_D^{25.4}$= +18.2 (c=1.00, $CHCl_3$); Lit[2] $[\alpha]_D^{26}$= +18.7 (c= 1.1, $CHCl_3$).

^{1}H NMR (500 MHz, $CDCl_3$): δ 1.36 (3H, d, *J*= 6.0 Hz), 4.31 (1H, dd, *J*= 2.0 Hz, 9.5 Hz), 5.57 (1H, m), 5.34 (1H, t, J= 9.5 Hz), 5.66 (2H, d, J= 4.5 Hz), 7.28-7.35 (3H, m), 7.47-7.53 (6H, m), 7.61 (1H, m), 8.1 (1H, d, *J*= 7.5 Hz).

^{13}C NMR (125 MHz, $CDCl_3$): δ 17.7, 67.7, 69.8, 75.0, 75.8, 86.0, 127.9, 128.6, 128.7, 129.3, 129.5, 129.5, 130.0, 130.0, 131.9, 133.7, 166.0, 167.2.

HRMS: (ESI-TOF, m/z) calcd for $C_{26}H_{24}O_6SNa$ 464.1294 found 464.1292.

1,2,4,5-Di-*O*-isopropylidene-3-*O*-naphth-2-ylmethyl-D-ribitol (19)

1,2,4,5-Di-*O*-isopropylidene ribitol (**18** [3], 1.5 g, 6.5 mmol) was dissolved in DMF (30 mL). 2-(bromomethyl)naphthalene (2.2 g, 9.75 mmol) was added, and the temperature was lowered to 0 °C and sodium hydride (0.52 g, 13 mmol) was added in parts. The reaction mixture was allowed to attain room temperature and then stirred overnight. TLC at this point showed complete conversion of reactant to product. The solvent was removed under reduced pressure. The crude residue was dissolved in CH_2Cl_2 (40 mL) and washed with H_2O (250 mL × 1). The aqueous layer was extracted with CH_2Cl_2 (25 mL × 4) and the combined organic layer was dried over anhydrous Na_2SO_4. The CH_2Cl_2 was removed under reduced pressure and the crude residue was purified by column chromatography (2.5% EA/PE) to give **19** (2.24 g, 93%) as a syrup.

^{1}H NMR (300 MHz, $CDCl_3$): δ 1.36 (6H, s), 1.45 (6H, s), 3.81-3.90 (2H, m), 3.93-3.98 (2H, m), 4.04-4.09 (2H, m), 4.19 (1H, dd, *J*= 6.4 Hz, 11.6 Hz), 4.95 (1H, s).

^{13}C NMR (75 MHz, $CDCl_3$): δ 25.1, 26.5, 30.9, 65.8, 74.8, 76.1, 78.5, 109.2, 125.9, 126.1, 126.1, 12.3, 126.6, 127.7, 127.9, 128.2, 133.0, 133.3, 135.7.

HRMS: (ESI-TOF, *m/z*) calcd for $C_{22}H_{28}O_5Na$ 395.1834 found 395.1833.

1,2,4,5-Tetra-*O*-benzyl-3-*O*-naphth-2-ylmethyl-D-ribitol (20)

1,2,4,5-Di-*O*-isopropylidene-3-*O*-naphthylmethyl-D-ribitol (**19**, 2.32 g, 6.23 mmol) was dissolved in MeOH (20 mL) and pTSA (6.23 mmol, 1.07 g) was added at room temperature. The reaction mixture was refluxed for 4 h. TLC at this point showed complete conversion of reactant to the corresponding deisopropylidenated product. The reaction mixture was poured into $NaHCO_3$ (aq.) (200 mL × 1) and washed. The aqueous layer was extracted with CH_2Cl_2 (50 mL × 4) and the combined organic layer was dried over anhydrous Na_2SO_4. The CH_2Cl_2 was removed under reduced pressure and the crude residue was co-evaporated with dry toluene (15 mL × 3). The dried residue was dissolved in DMF (40 mL) and benzyl bromide (3.6 mL, 30 mmol) was added. The temperature was lowered to 0 °C and sodium hydride (1.52 g, 30 mmol) was added in parts. The reaction mixture was allowed to attain room temperature and then stirred overnight. TLC at this point showed complete conversion of the reactant to product. The solvent was removed under reduced pressure. The crude residue was dissolved in CH_2Cl_2 (40 mL) and washed with H_2O (300 mL × 1). The aqueous layer was extracted with CH_2Cl_2 (25 mL × 4) and the combined organic layer was dried over anhydrous Na_2SO_4. The CH_2Cl_2 was removed under reduced pressure and the crude residue was purified by column chromatography (20% EA/PE) to give **20** (3.86 g, 95%) as colorless syrup.

$[\alpha]_D^{25.4}$= -0.031 (c=1.53, $CHCl_3$).

^{1}H NMR (300 MHz, $CDCl_3$): δ 3.72-3.75 (4H, m), 3.95-3.99 (3H, m), 4.49 (4H, s), 4.60-4.75 (5H, m), 4.82 (1H, s), 7.26-7.48 (22H, m), 7.66 (1H, s), 7.75-7.82 (4H, m).

^{13}C NMR (75 MHz, $CDCl_3$): δ 70.2, 72.4, 73.3, 73.8, 77.2, 78.6, 78.7, 125.8, 125.9, 126.2, 126.7, 127.4, 127.5, 127.7, 127.8, 127.9, 127.9, 128.3, 128.3, 132.9, 133.3, 135.9, 138.5, 138.7.

HRMS: (ESI-TOF, *m/z*) calcd for $C_{44}H_{44}O_5Na$ 675.3086 found 675.3085.

1,2,4,5-Tetra-*O*-benzyl-D-ribitol (7) [4]

1,2,4,5-Tetra-*O*-benzyl-3-*O*-naphthylmethyl-D-ribitol (**20**, 1.13 g, 1.73 mmol) was dissolved in CH_2Cl_2/H_2O (19:1) (20 mL) and DDQ (473 mg, 20.8 mmol) was added. The reaction mixture was allowed to stir at room temperature for 2 h. TLC at this point showed complete conversion of reactant to product. The reaction mixture was diluted with CH_2Cl_2(40 mL) and the organic layer was washed with $NaHCO_3$ (aq) (200 mL × 1). The aqueous layer was extracted with CH_2Cl_2(25 mL × 4) and the combined organic layer was dried over anhydrous Na_2SO_4. The $CH_2Cl_2$was removed under reduced pressure and the crude residue was purified by column chromatography (10% EA/PE) to give **7** (0.67 g, 85%) as yellow oil.

$[\alpha]_D^{25.4}$= -+4.7 (c=1.03, $CHCl_3$); Lit[4] $[\alpha]_D^{23}$= +4.3 (c= 1.0, $CHCl_3$).

^{1}H NMR (300 MHz, $CDCl_3$): δ 3.73- 3.85 (6H, m), 4.15 (1H, m), 4.58-4.78 (8H, m), 7.36-7.39 (20H, m).

^{13}C NMR (75 MHz, $CDCl_3$): δ 70.2, 71.7, 72.1, 73.6, 78.2, 127.7, 127.8, 127.8, 128.0, 128.4, 128.5, 138.2, 138.5.

HRMS: (ESI-TOF, m/z) calcd for $C_{33}H_{36}O_5Na$ 535.2460 found 535.2459.

General procedures for the various glycosylation protocols followed in the overall synthesis

Method A (BSP, Tf_2O): The donor (1.0 equiv) and BSP (1.1 equiv) were dissolved in CH_2Cl_2 and then stirred with 4Å MS at room temperature for 10 mins. The reaction temperature was lowered to −60 °C and Tf_2O (1.02 equiv) was added and then stirred for 15 mins at the same temperature. The acceptor (1.0 equiv) was added next at that temperature and then the reaction temperature was raised to room temperature over 1 h. TLC at this point indicated reaction status. Upon completion of the reaction, standard work up involving filtration of 4 Å MS over celite bed, followed by $NaHCO_3$ (aq) wash and brine wash of the filtrate, drying over anhydrous Na_2SO_4. Finally column chromatographic purification was carried out as applicable to isolate products.

Method B (Ph_2SO, Tf_2O): The donor (1.2–1.5 equiv) was dissolved in CH_2Cl_2. Ph_2SO (2.2 equiv with respect to donor) and TTBP (1.0 equiv) was added and the temperature was lowered to −60 °C and Tf_2O (1.02 equiv to donor) was added and then stirred for 5 min at the same temperature. The temperature was raised to −40 °C and stirred at this temperature for 1 h. The acceptor (1.0 equiv) was added as a solution in CH_2Cl_2. The temperature was then raised to room temperature and stirring was continued till reaction completion as indicated by TLC. Once complete the reaction mixture was washed with $NaHCO_3$ (aq) and then brine and then the filtrate was dried over anhydrous Na_2SO_4. Finally column chromatographic purification was carried out as applicable to isolate products.

Method C (TMSOTf): The trichloroacetimidate donor (1.0–1.4 equiv) and acceptor (1.0 equiv) was dissolved in the selected solvent and then stirred with 4 Å MS at room temperature for 10 min. The reaction temperature was set up as required and then TMSOTf (0.3 equiv to donor) was added and the reaction was allowed to continue at the temperature set up. TLC was used to indicate reaction status and upon completion the 4 Å MS was filtered under suction. Standard work up of the filtrate involving $NaHCO_3$ (aq) followed by brine wash and then the filtrate was dried over anhydrous Na_2SO_4. Finally column chromatographic purification was carried out as applicable to isolate products.

Method D (NIS/TMSOTf): The donor (1.0–1.4 equiv) and acceptor (1.0 equiv) was dissolved in the selected solvent and then stirred with 4 Å MS at room temperature for 10 min. The reaction temperature was set up as required. NIS (1.0–2.0 equiv) and TMSOTf (0.3 equiv to donor) were added and the reaction was allowed to continue at the temperature set up. TLC was used to indicate reaction status and upon completion the 4 Å MS was filtered under suction. Standard work up involving successive $NaHCO_3$ (aq) and $Na_2S_2O_3$ (aq) wash followed by drying over anhydrous Na_2SO_4 were carried out. Finally column chromatographic purification was done as applicable to isolate products.

Phenyl 2-*O*-benzyl-4,6-*O*-benzylidene-3-*O*-napth-2-ylmethyl-α-D-glucopyranosyl-(1→3)-2,4-di-*O*-benzoyl-1-thio-α-L-rhamnopyranoside (3a)

Donor **6b** (0.48 g, 0.75 mmol) and acceptor **5** (0.26 g, 0.56 mmol) were coupled using method **C** in the presence of TMSOTf (41 μL) ether/CH_2Cl_2 (1:5) and then purified by column chromatography (12% EA/PE) to give the disaccharide **3a** (399 mg, 75%) as white foam.

$[\alpha]_D^{25.4}$= +18.59 (c= 2.83, $CHCl_3$).

^{1}H NMR (300 MHz, $CDCl_3$):δ 1.36 (3H, d, *J*= 6.18 Hz), 3.38-3.48 (3H, m), 3.69-3.89 (3H, m), 4.34 (1H, m, *H*-3), 4.53-4.71 (4H, m, H-5, Bn& Nap-*H*), 4.89 (1H, d, *J*= 3.3 Hz, *H*-1), 5.35 (1H, s, PhC*H*), 5.68 (1H, t, *J*= 9.7 Hz, *H*-4), 5.74 (1H, bs, *H*-1), 5.86 (1H, bd, *J*= 1.6 Hz, *H*-2), 7.21-7.67 (27H, m), 7.79 (1H, m, Ar*H*), 8.05-8.13 (4H, m, Ar*H*).

^{13}C NMR (75 MHz, $CDCl_3$):δ 17.6 (CH_3), 63.3, 68.2, 71.4, 72.9, 73.0, 73.8, 75.1, 78.2, 78.4, 81.6, 86.0 (C-1), 96.8 (C-1’), 101.2 (Ph*C*H), 125.8, 126.1, 126.3, 127.6, 127.8, 127.9, 127.9, 128.0, 128.3, 128.5, 128.6, 129.2, 129.7, 130.1, 131.8, 133.3, 133.4, 137.5, 165.7 (*C*=O), 166.1 (*C*=O).

HRMS: (ESI-TOF, *m/z*) calcd for $C_{57}H_{52}O_{11}SNa$ $[M + Na]^+$ 967.3128 found 967.3122.

Anal. Calcd for $C_{57}H_{52}O_{11}S$ C = 72.44, H = 5.55, S = 3.39; found C =72.36, H = 5.48, S = 3.37.

Phenyl 2-*O*-benzyl-4,6-*O*-benzylidene-α-D-glucopyranosyl-(1→3)-2,4-di-*O*-benzoyl-1-thio-α-L-rhamnopyranoside (4)

Disaccharide **3a** (240 mg, 0.25 mmol) was dissolved in 9:1 CH_2Cl_2/H_2O (10 mL) and DDQ (86 mg, 0.38 mmol) was added and the reaction mixture was stirred at room

temperature for 2 h. TLC analysis at this point showed the reaction to be complete. The reaction mixture was diluted with CH_2Cl_2 (20 mL) and washed with $NaHCO_3$ (aq) (100 mL × 1). The aqueous layer was extracted with CH_2Cl_2 (10 mL × 4).The combined organic layer was dried over anhydrous Na_2SO_4. The CH_2Cl_2 was removed under reduced pressure. The crude product was purified by column chromatography (20% EA/PE) to give the product **4** (190 mg, 93%) as white foam.

$[\alpha]_D^{25.5}$= +17.34 (c= 2.83, $CHCl_3$).

^{1}H NMR (300 MHz, $CDCl_3$): δ 1.27 (3H, d, *J*= 6.3 Hz), 2.15 (1H, bs, O*H*), 3.18-3.27 (2H, m, *H*-2’, *H*-4’), 3.41 (1H, t, *J*= 10.2 Hz, *H*-6b’), 3.62 (1H, dt, *J*= 4.8 Hz, 9.9 Hz, *H*-5’), 3.80 (1H, t, *J*= 9.3 Hz, *H*-3’), 3.92 (1H, dd, *J*= 4.8 Hz, 10.2 Hz, *H*-6b’), 4.26-4.33 (2H, m, *H*-3, Bn-*H*), 4.42-4.49 (2H, m, *H*-5, Bn-*H*), 4.93 (1H, d, *J*= 3.6 Hz, *H*-1’), 5.22 (1H, s, PhC*H*), 5.56-5.63 (2H, m, *H*-1, *H*-4), 5.78 (1H, dd, *J*= 1.8 Hz, 3.0 Hz, *H*-2), 7.05-7.51 (24H, m, Ar*H*), 8.00-8.05 (4H, m, Ar*H*).

^{13}C NMR (75 MHz, $CDCl_3$): δ 17.7 (CH_3), 62.9, 68.3, 68.7, 69.9, 70.9, 72.7, 72.8, 73.0, 78.7, 80.9, 86.1 (C-1), 95.3 (C-1’), 101.9 (Ph*C*H), 126.6, 127.9, 128.0, 128.1, 128.6, 128.7, 128.8, 129.1, 129.4, 129.5, 129.8, 130.1, 131.9, 133.5, 133.5, 133.6, 137.2, 137.8, 165.7 (*C*=O), 166.2 (*C*=O).

HRMS: (ESI-TOF, *m/z*) calcd for $C_{46}H_{44}O_{11}SNa$ $[M + Na]^+$ 827.2502 found 827.2499.

Anal. Calcd for $C_{46}H_{44}O_{11}S$: C = 68.64, H = 5.51, S = 3.98; found C =68.81, H = 5.46, S = 3.97.

Phenyl 2,3,4,6-tetra-*O*-benzyl-α-D-galactopyranosyl-(1→3)-2-*O*-benzyl-4,6-O-benzylidene-α-D-glucopyranosyl-(1→3)-2,4-di-*O*-benzoyl-1-thio-α-L-rhamnopyranoside (21)

Donor **2** (0.21g 0.31 mmol) and acceptor **4** (0.21 g, 0.26 mmol) were coupled in the presence of TMSOTf (18 μL) in ether/CH_2Cl_2 (1:4) by method C and purified by column chromatography (10% EA/PE) to give the trisaccharide **21**(0.22 g, 70%) as white foam.

$[\alpha]_D^{24.1}$= +55.14 (c= 1.12, $CHCl_3$).

^{1}H NMR (300 MHz, $CDCl_3$): δ 1.41 (3H, d, *J*= 6.0 Hz, C*H*$_3$), 3.46-3.65 (5H, m), 3.75-3.85, (2H, m) 3.88-3.96 (2H, m), 4.06 (1H, dd, *J*= 5.4, 10.8 Hz), 4.14 (1H, t, *J*= 9.4 Hz), 4.25 (1H, m), 4.29-4.42 (4H, m), 4.47-4.56 (5H, m), 4.68 (1H, d, *J*= 11.7 Hz, Bn-*H*), 4.79 (1H, d, *J*= 11.8 Hz, Bn-*H*), 4.87 (1H, d, *J*= 11.2 Hz, Bn-*H*), 5.00 (1H, d, *J*= 3.2 Hz, *H*-1′), 5.32 (1H, s, PhC*H*), 5.41 (1H, d, *J*= 3.5 Hz, *H*-1″), 5.71-5.78 (2H, m, *H*-1, *H*-4), 5.90 (1H, bs, *H*-2), 7.01-7.61 (41H, m, Ar*H*), 8.11-8.16 (4H, m, Ar*H*).

^{13}C NMR (75 MHz, $CDCl_3$): δ 17.7 (*C*H$_3$), 62.9, 67.8, 68.2, 68.3, 68.8, 71.5, 71.7, 71.7, 72.8, 73.0, 73.1, 73.2, 73.7, 75.0, 75.1, 75.8, 77.7, 78.4, 82.7, 86.0 (*C*-1), 96.2 & 96.6 (*C*-1’ &*C*-1’’), 101.7 (Ph*C*H), 126.5, 127.3, 127.4, 127.4, 127.6, 127.9, 127.9, 128.0, 128.1, 128.2, 128.2, 128.3, 128.4, 128.6, 128.8, 129.0, 129.2, 129.4, 129.5, 129.6, 129.8, 130.1, 131.9, 133.4, 133.6, 133.7, 137.2, 137.8, 138.5, 138.8, 139.1, 139.2, 165.7 (*C*=O), 166.1 (*C*=O).

HRMS: (ESI-TOF, *m/z*) calcd for $C_{80}H_{78}O_{16}SNa$ $[M + Na]^+$ 1349.4908 found 1349.4906.

Anal. Calcd for $C_{80}H_{78}O_{16}S$; C = 72.38, H = 5.92, S = 2.41; found C =72.43, H = 5.89, S = 2.40.

2,3,4,6-Tetra-*O*-benzyl-α-D-galactopyranosyl-(1→3)-2-*O*-benzyl-4,6-*O*-benzylidene-α-D-glucopyranosyl-(1→3)-2,4-di-*O*-benzoyl-α-L-rhamnopyranosyl-(1→3)-1,2,4,5-tetra-*O*-benzyl-D-ribitol (1)

Donor **21** (0.20g 0.17 mmol) and acceptor **7**(0.09 g, 0.14 mmol) were coupled in the presence of TMSOTf (6 µL) in CH_2Cl_2 by method **D** to give the tetrasaccharide **1** (0.24 g, 89%) as syrup.

$[\alpha]_D^{27.1}$= +85.14 (c= 1.12, $CHCl_3$)

^{1}H (300 MHz, $CDCl_3$): δ 1.12 (3H, d, J= 6.0 Hz, CH_3), 3.49-3.54 (6H, m), 3.70-3.85 (9H, m), 3.95-4.05 (3H, m), 4.22-4.35 (8H, m), 4.43-4.71 (13H, m), 4.78 (1H, m), 5.02 (1H, d, *J*=3.2 Hz, *H*-1″),5.25 (1H, apt, *J*=7.2 Hz, *H*-4′), 5.31 (1H, d, *J*=3.6 Hz, *H*-1′), 5.51-5.62 (2H, m,*H*-2′, PhC*H*), 5.57-5.59 (2H, m), 6.94-7.11 (10H, m, Ar*H*), 7.18-7.45 (46H, m, Ar*H*), 8.01-8.07 (4H, m, Ar*H*).

^{13}C (75 MHz, $CDCl_3$): δ 17.8 (CH_3), 60.5, 62.6, 67.6, 67.7, 68.1, 68.7, 68.8, 69.3, 70.2, 71.5, 71.7, 72.2, 72.6, 72.7, 72.8, 73.0, 73.3, 73.3, 74.9, 75.1, 75.7, 76.9, 77.3, 78.0, 78.4, 82.8, 94.9 & 96.5 (C-1 & C-1″), 97.7 (C-1′), 101.6 (Ph*C*H), 126.5, 127.2, 127.4, 127.4, 127.6, 127.7, 127.8, 127.8, 127.9, 127.9, 128.1, 128.2, 128.3, 128.3, 128.4, 128.5, 128.6, 128.7, 129.1, 129.6. 129.6, 129.8, 129.9, 130.1, 133.2, 133.4, 137.2, 137.7, 138.3, 138.5, 138.8, 139.0, 139.1, 139.4, 165.7 (*C*=O), 166.2 (*C*=O).

HRMS: (ESI-TOF, m/z) calcd for $C_{107}H_{108}O_{21}SNa$ [M + Na]+ 1751.7281 found 1751.7280.

Anal. Calcd for $C_{107}H_{108}O_{21}$: C = 74.29, H = 6.29; found C =74.13, H = 6.33.

One-pot synthesis of the protected tetrasaccharide 1

Donor **2** (85 mg, 0.12 mmol) and acceptor **4** (75 mg, 0.09 mmol) were dissolved in ether/CH_2Cl_2 (1:4) (20 mL) and stirred at room temperature with 4 Å MS for 10 mins. The temperature was lowered to −15 °C. TMSOTf (7 μL, 0.04 mmol) was added and the reaction was continued for 1 h. On completion of the first step as indicated by TLC, second acceptor **7** (41.5 mg, 0.08 mmol) followed by NIS (58 mg, 0.24 mmol) was added at that temperature. And then TMSOTf (4 μL, 0.02 mmol) was added to this reaction mixture. The temperature was raised to −10 °C and the reaction was stirred at that temperature for 45 min. TLC at this point showed optimum conversion of reactants to product. The MS was filtered over a celite bed and the filtrate obtained was washed with $Na_2S_2O_3$ (aq) (100 mL × 1) and $NaHCO_3$ (aq) (100 mL × 1) in succession. The aqueous layers were extracted with CH_2Cl_2 (25 mL × 5) and the combined organic layer was dried over anhydrous Na_2SO_4. The CH_2Cl_2 was removed under reduced pressure and the crude mass was purified by column chromatography (15% EA/PE) to give the product **1** (77 mg, 52%). The analytical data obtained was in agreement with the product obtained from the step wise synthesis.

α-D-Galactopyranosyl-(1→3)-α-D-glucopyranosyl-(1→3)-α-L-rhamnopyranosyl-(1→3)-D-ribitol (23) [4]

Compound **1** (90 mg, mmol) was dissolved in NaOMe/MeOH solution 0.1 M (15 mL) and then stirred for 15 h at room temperature. It was then quenched with Dowex 50W resin and then filtered. The solvent was removed under reduced pressure and the crude mass was dissolved in EtOH/EA/AcOH (1:1:0.1) (5 mL). 10% Pd-C (25 mg) was added

and the reaction mixture was stirred in an atmosphere of H_2 at room temperature for 20 h. The catalyst was filtered off, washed with MeOH. The filtrate was concentrated to give the foamy solid product **23** (27.5 mg, 85%).

$[\alpha]_D^{24.1}$= +77.9 (c= 2.75, H_2O); Lit[4] $[\alpha]_D^{26}$= +78.2 (c= 0.8, H_2O).

^{1}H NMR (500 MHz, D_2O): δ 1.22 (3H, d, *J*= 6.0 Hz, *H*-6′), 3.50 (1H, t, *J*= 9.5 Hz H-4'), 3.53-3.57 (2H, m),3.59-3.61 (2H, m, H-2'') 3.63-3.65 (3H, m), 3.66-3.72 (4H, m, H-3'', H-6''', H-5'), 3.73-3.79 (3H, m, H-3', H-2'''), 3.81-3.86 (3H, m, H-3'''), 3.87-3.93 (3H, m, H-4''', H-5''), 4.12 (1H, m, H-2'), 4.17 (1H, app. t, H-5'''), 4.93 (1H, s, H-1'), 5.02 (1H, d, *J*= 3.0 Hz, H-1''), 5.31 (1H, d, *J*= 3.5 Hz, H-1''').

^{13}C NMR (125 MHz, D_2O):δ 16.6 (*C*-6'), 60.0 (C-6'''), 60.8, 62.3, 62.6, 66.8 (C-2'), 68.6, 69.1 (C-4'''), 69.3, 69.4, 69.7 (C-2''), 70.0 (C-4'), 70.1, 70.7, 70.8, 71.4, 71.6, 75.2 (C-3'), 79.6 (C-3''), 79.8 (C-5'), 95.4 (*C*-1″), 99.2 (*C*-1‴), 100.1 (*C*-1′).

HRMS: (ESI-TOF, m/z) calcd for $C_{23}H_{42}O_{19}$ Na [M + Na]+ 645.2218 found 645.2212.

References

1. Crich, D.; Vinogradova, O. *J. Org. Chem.*, **2007**, *72*, 3581.
2. Rao, Yu. Boons, G-J. *Angew. Chem. Int. Ed.*.**2007**, *46,* 6148.
3. Xie, Z. F.; Suemune, H.; Sakai, K. *Tetrahedron: Asymetry,***1993**, *4*, 973.
4. Parameswar, A.; Pornsuriyasak, P.; Lubanowski, N. A.; Demchenko, A. V. *Tetrahedron*, **2007**,*63*, 10083.
