# Supplementary material for: Synthetic avenues towards a tetrasaccharide related to Streptococcus pneumonia of serotype 6A
Source: Beilstein J Org Chem. 2018 May 17;14:1095–102. doi: 10.3762/bjoc.14.95 (PMC6009338; doi:10.3762/bjoc.14.95)

# Supporting Information File 2

# for

# Synthetic avenues towards a tetrasaccharide related to *Streptococcus pneumonia* of serotype 6A

Aritra Chaudhury[1,2], Mana Mohan Mukherjee[1,3] and Rina Ghosh[1*]

Address:Department of Chemistry, Jadavpur University, 188, Raja S. C. Mullick Rd., Kolkata 700032, India, [2]present address: Department of Chemical Sciences, Indian Institute of Science Education and Research, Kolkata, Mohanpur, 741246, West Bengal, India and [3]present addrress: Laboratory of Bioorganic Chemistry, NIH, NIDDK, Bethesda, MD, USA

Email: Rina Ghosh[*]- ghoshrina@yahoo.com

* Corresponding author

## $^{1}$H and $^{13}$C NMR of compounds 1, 3a, 4, 5, 6a, 6b, 7, 12a, 19, 20, 21, and 23 and 2D NMR (COSY, HSQC and HMBC) of compound 23

| | |
|---|---|
| Contents | S1 |
| $^{1}$H NMR and $^{13}$C NMR spectra of compound **6a** | S2 |
| $^{1}$H NMR and $^{13}$C NMR spectra of compound **12a** | S3 |
| $^{1}$H NMR and $^{13}$C NMR spectra of compound **16** | S4 |
| $^{1}$H NMR and $^{13}$C NMR spectra of compound **19** | S5 |
| $^{1}$H NMR and $^{13}$C NMR spectra of compound **20** | S6 |
| $^{1}$H NMR and $^{13}$C NMR spectra of compound **3a** | S7 |
| $^{1}$H NMR and $^{13}$C NMR spectra of compound **4** | S8 |
| $^{1}$H NMR and $^{13}$C NMR spectra of compound **21** | S9 |
| $^{1}$H NMR and $^{13}$C NMR spectra of compound **1** | S10 |
| $^{1}$H NMR and $^{13}$C NMR spectra of compound **23** | S11 |
| $^{1}$H-$^{13}$C Non-decoupled spectra of compound **23** | S12 |
| $^{1}$H-$^{1}$H COSY spectra and expansion for compound **23** | S13 |
| $^{1}$H-$^{13}$C HSQC spectra and expansion for compound **23** | S14 |
| $^{1}$H-$^{13}$C HMBC spectra expansion for compound **23** | S15 |
| $^{1}$H-$^{13}$C HMBC spectra expansion for compound **23** | S15 |

$^{1}$H-NMR (300 MHz) spectra of compound **6a (CDCl$_3$)**

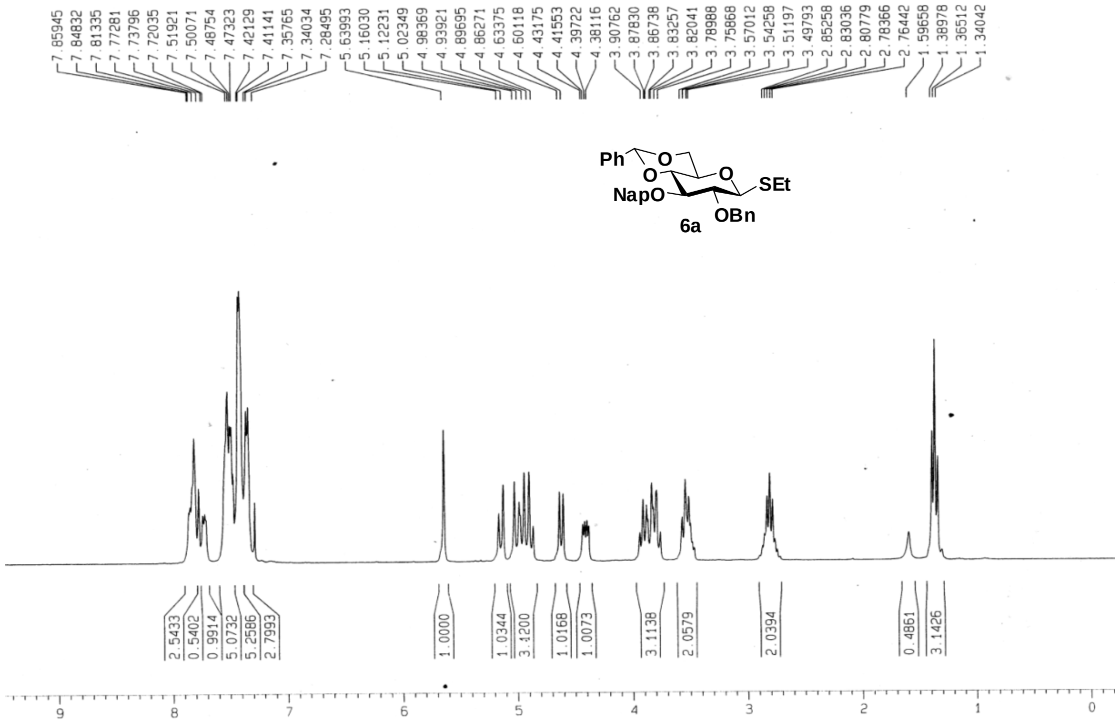


$^{13}$C-NMR (75 MHz) spectra of compound **6a (CDCl$_3$)**

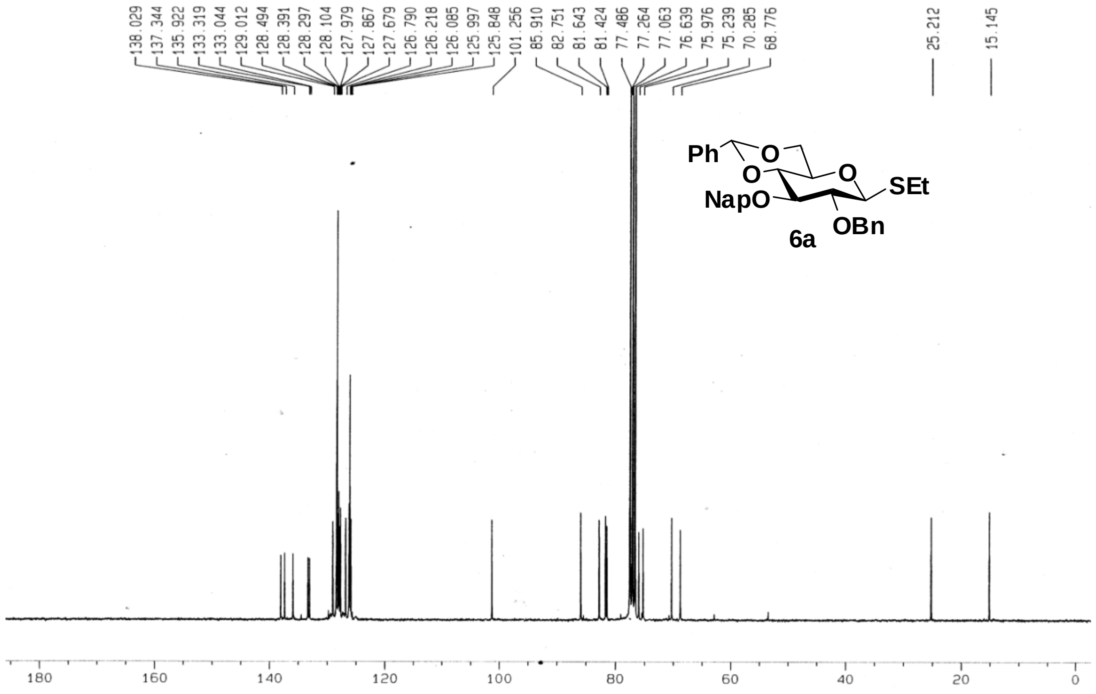

$^{1}$H-NMR (300 MHz) spectra of compound **12a (CDCl$_3$)**

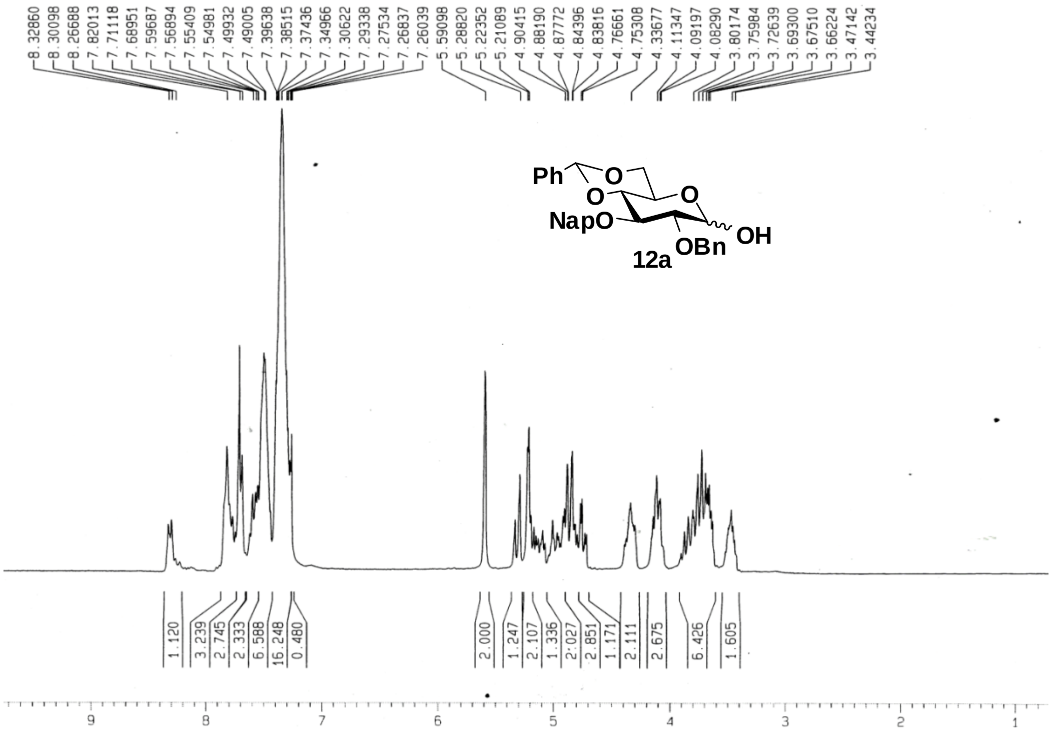


$^{13}$C-NMR (75 MHz) spectra of compound **13a (CDCl$_3$)**

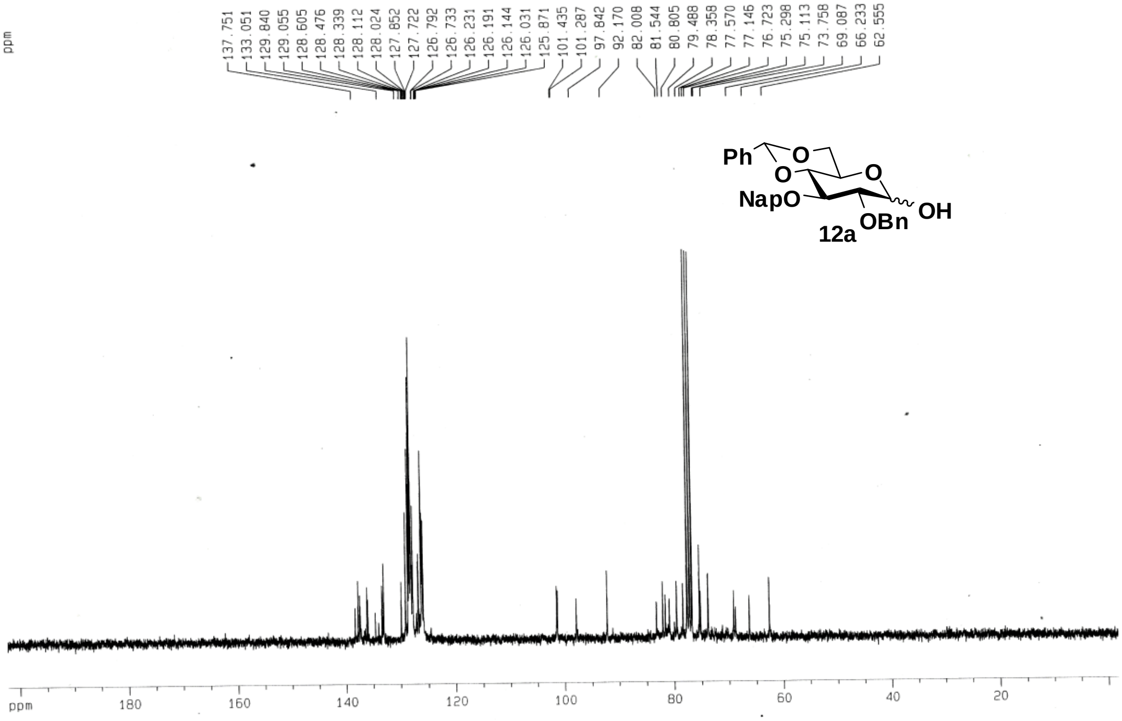

$^{1}$H-NMR (300 MHz) spectra of compound **16 (CDCl$_3$)**

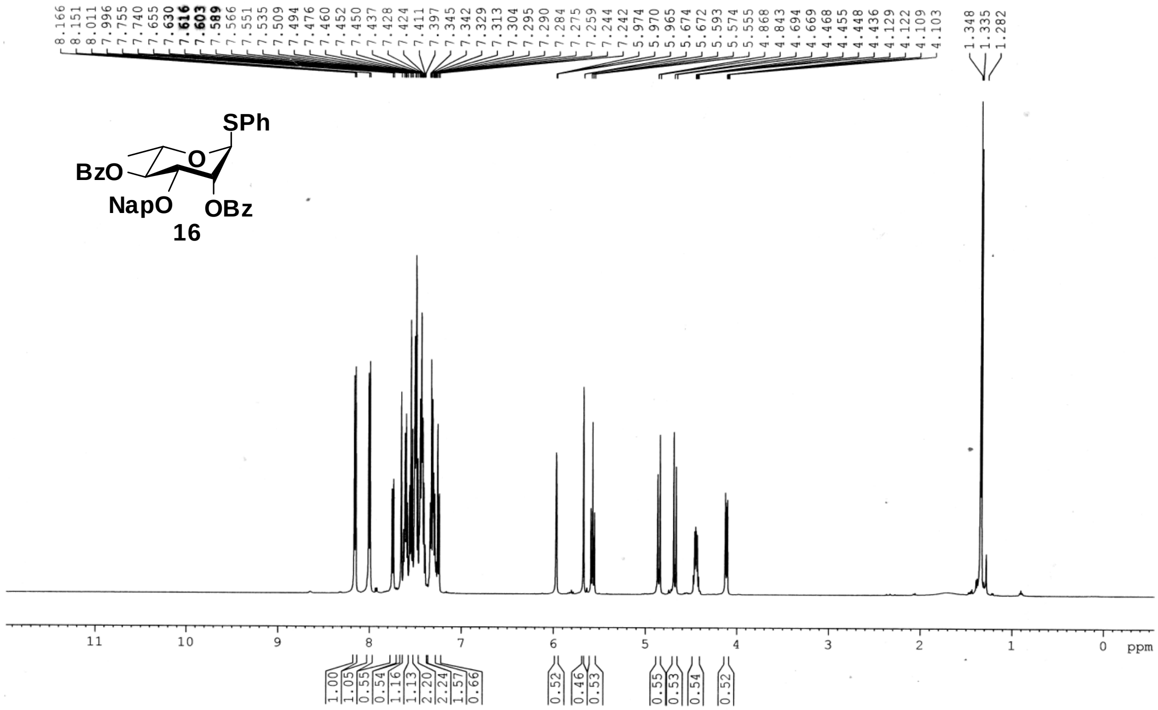


$^{13}$C-NMR (75 MHz) spectra of compound **16 (CDCl$_3$)**

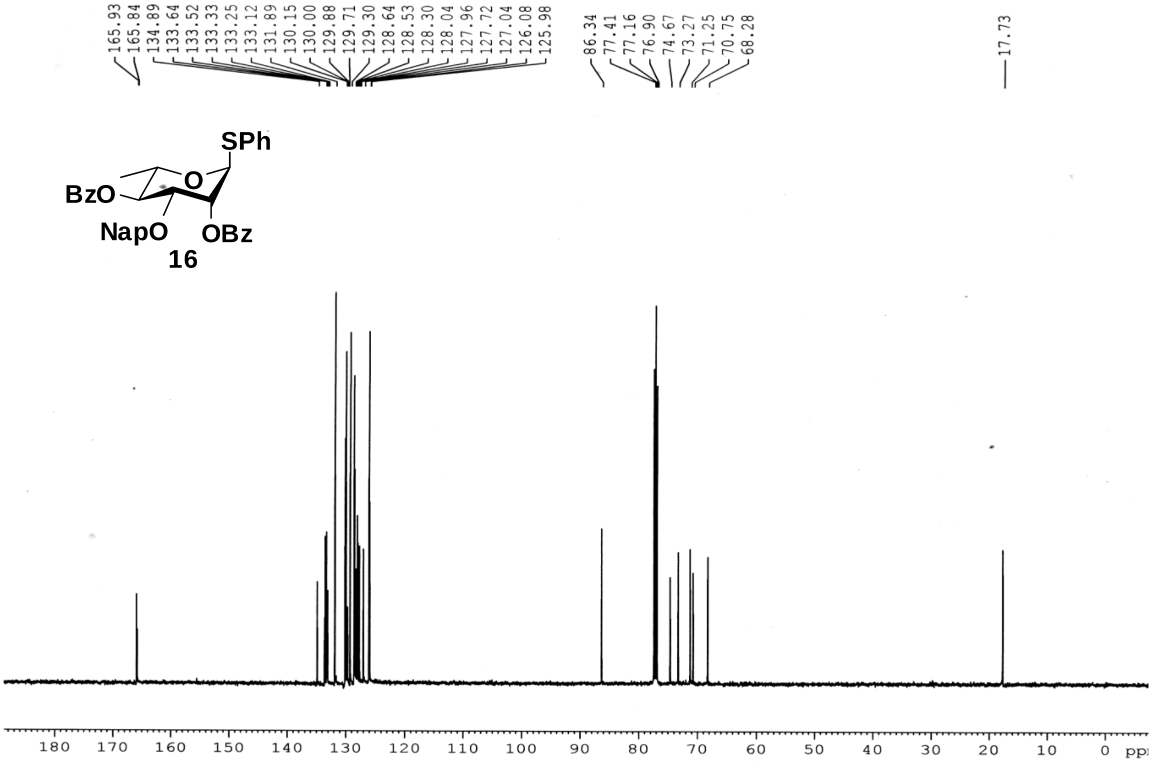

$^{1}$H-NMR (300 MHz) spectra of compound **19 (CDCl$_3$)**

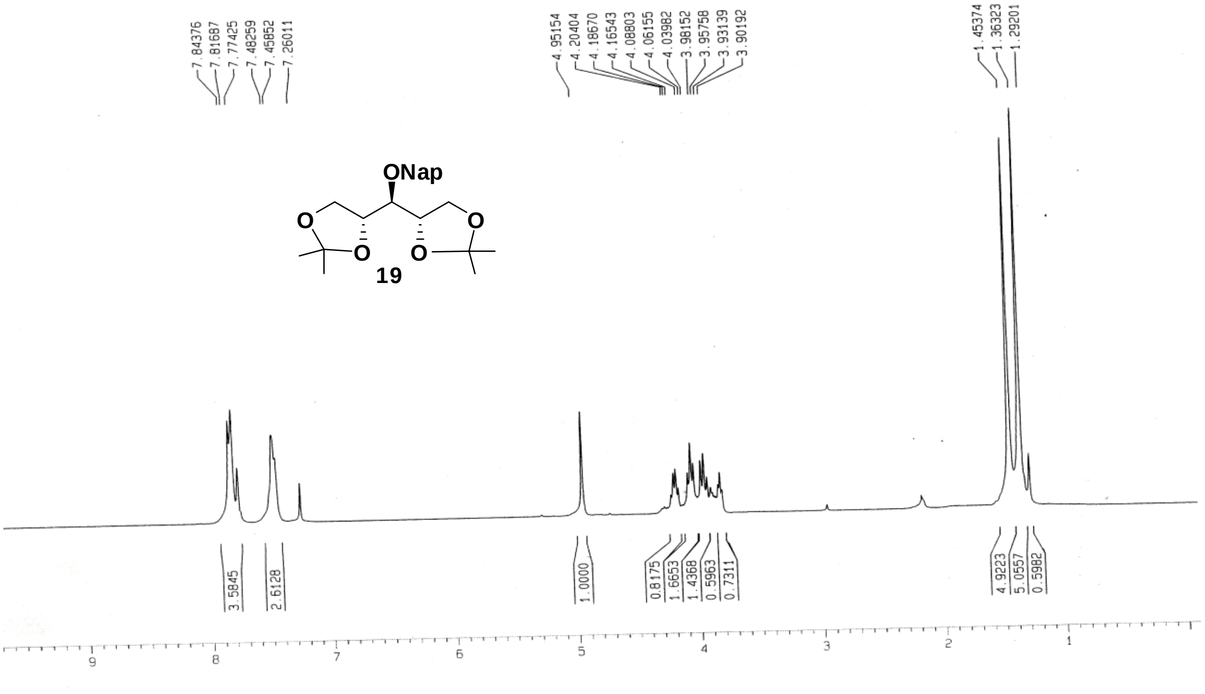


$^{13}$C-NMR (75 MHz) spectra of compound **19 (CDCl$_3$)**

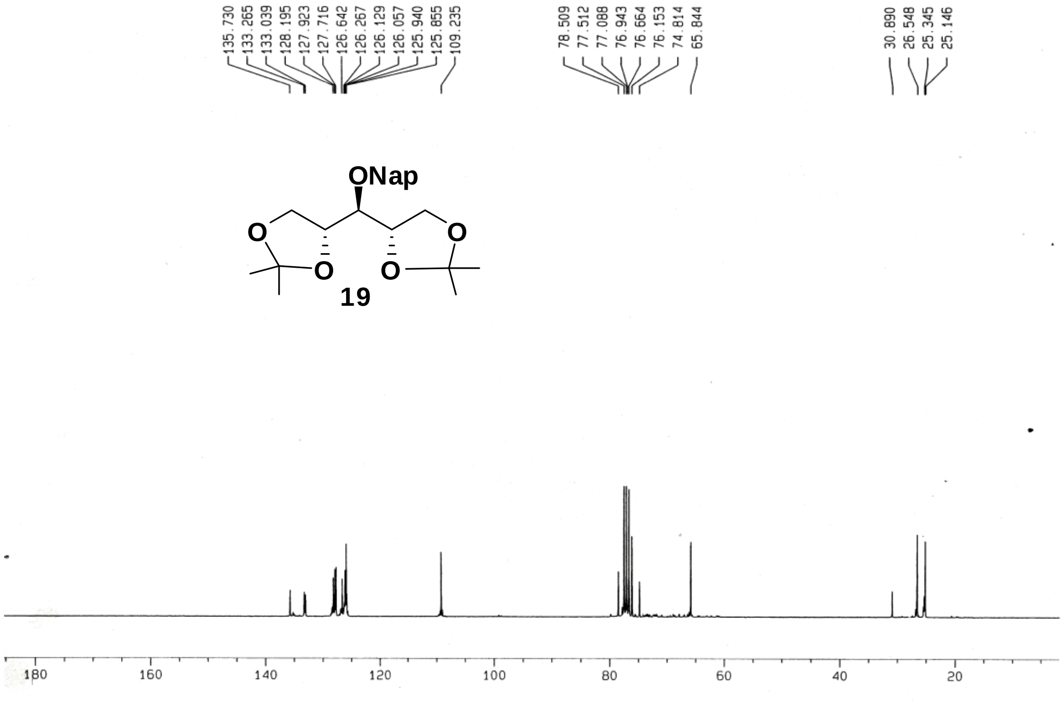

$^{1}$H-NMR (300 MHz) spectra of compound **20 ($CDCl_3$)**

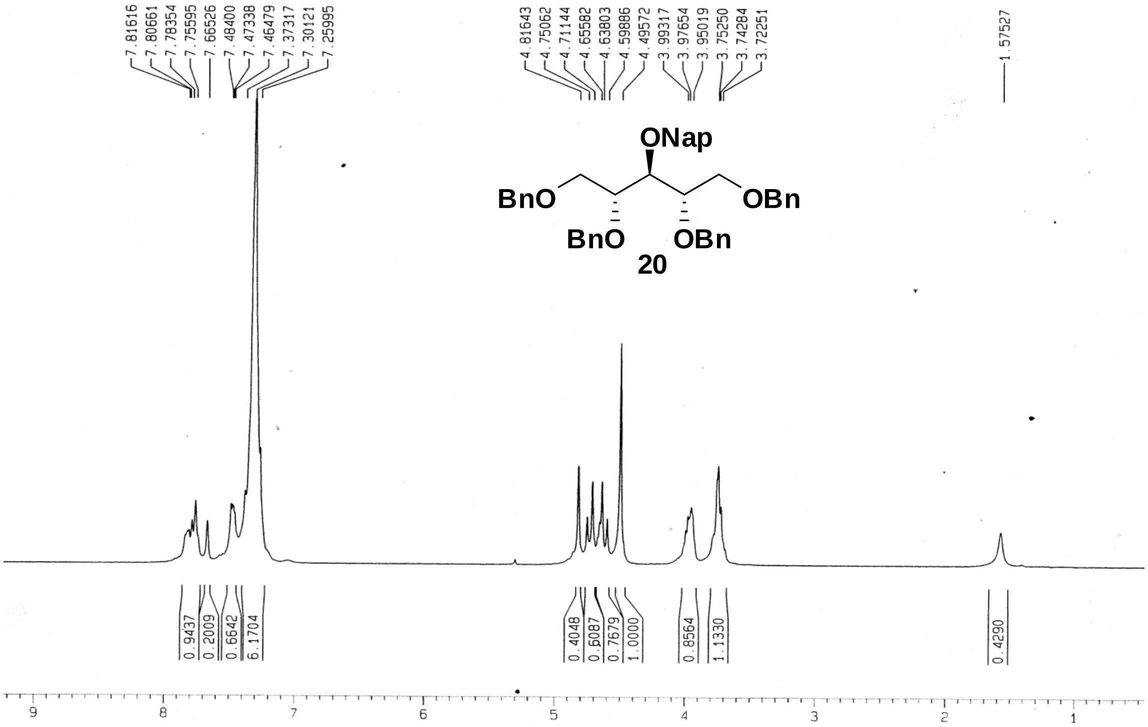


$^{13}$C-NMR (75 MHz) spectra of compound **20 ($CDCl_3$)**

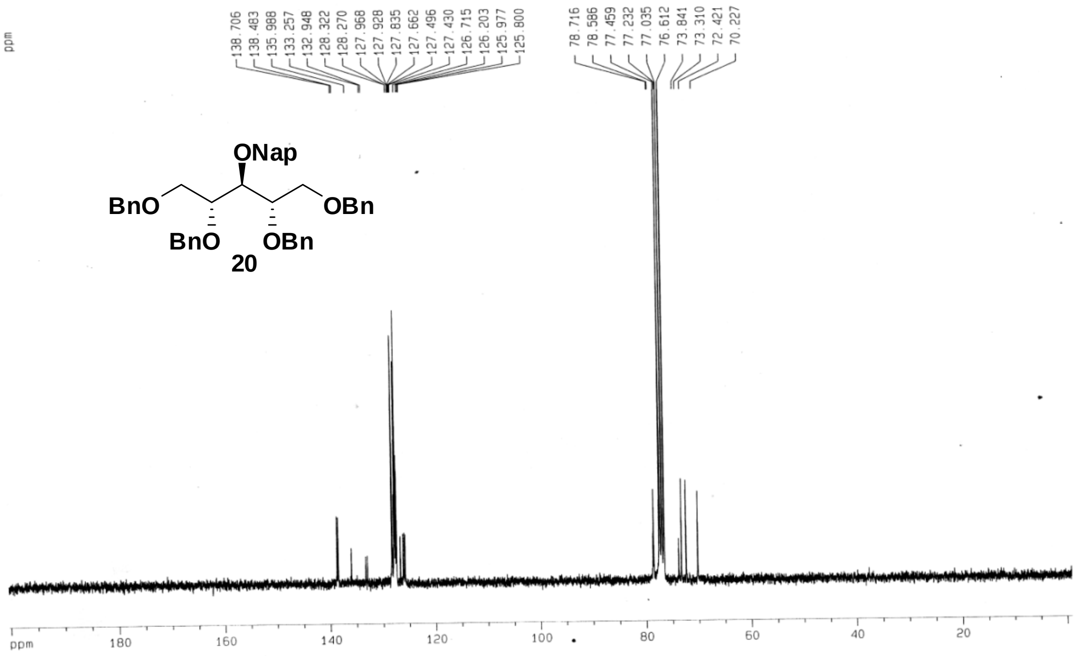

$^1$H-NMR (300 MHz) spectra of compound **3a (CDCl$_3$)**

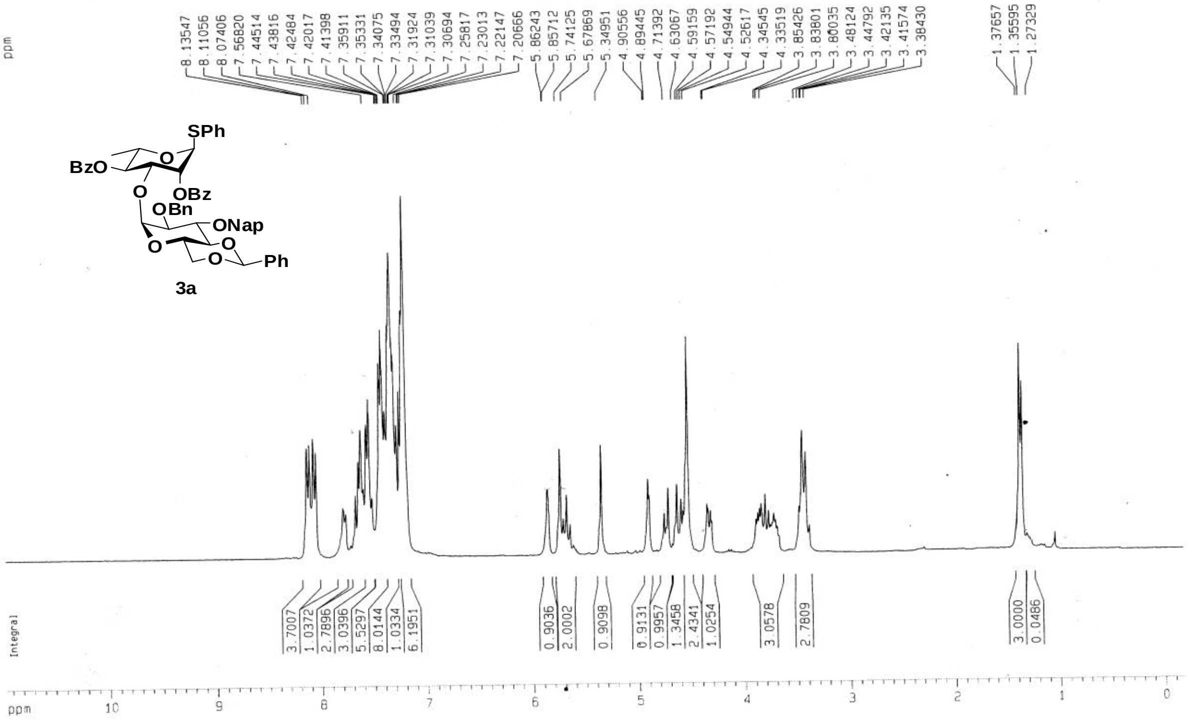

$^{13}$C-NMR (75 MHz) spectra of compound **3a (CDCl$_3$)**

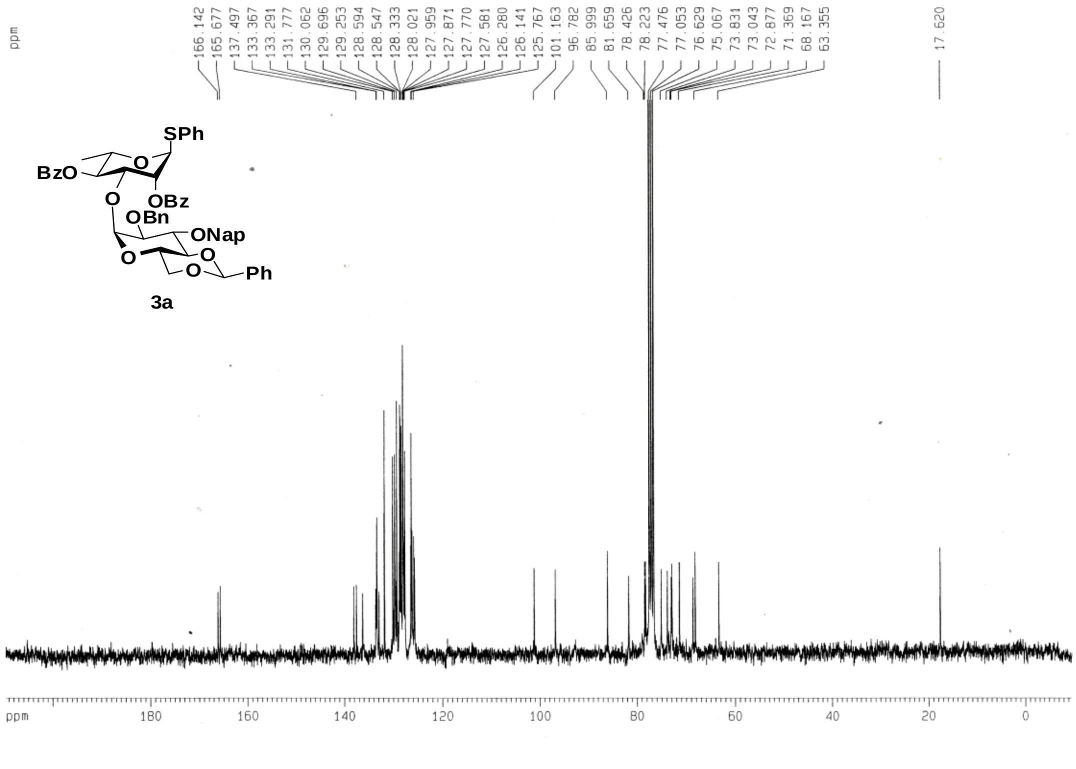

$^{1}$H-NMR (300 MHz) spectra of compound **4(CDCl$_3$)**

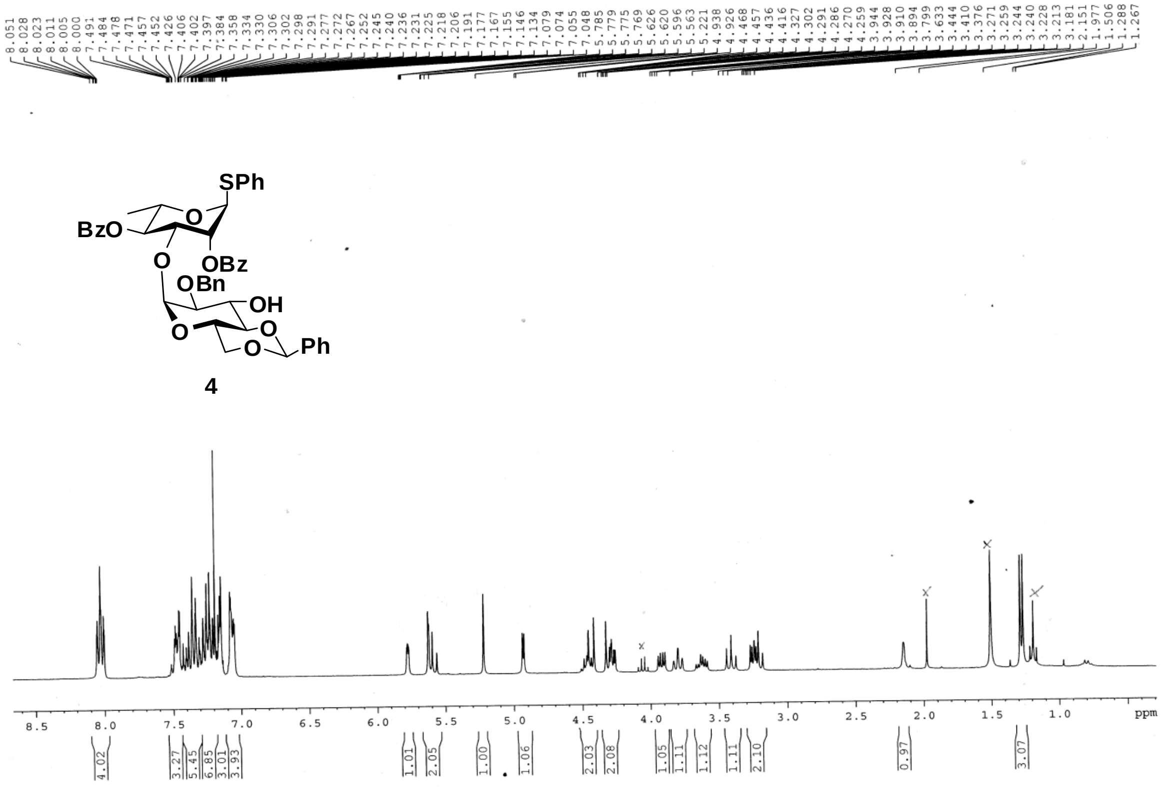


$^{13}$C-NMR (75 MHz) spectra of compound **4 (CDCl$_3$)**

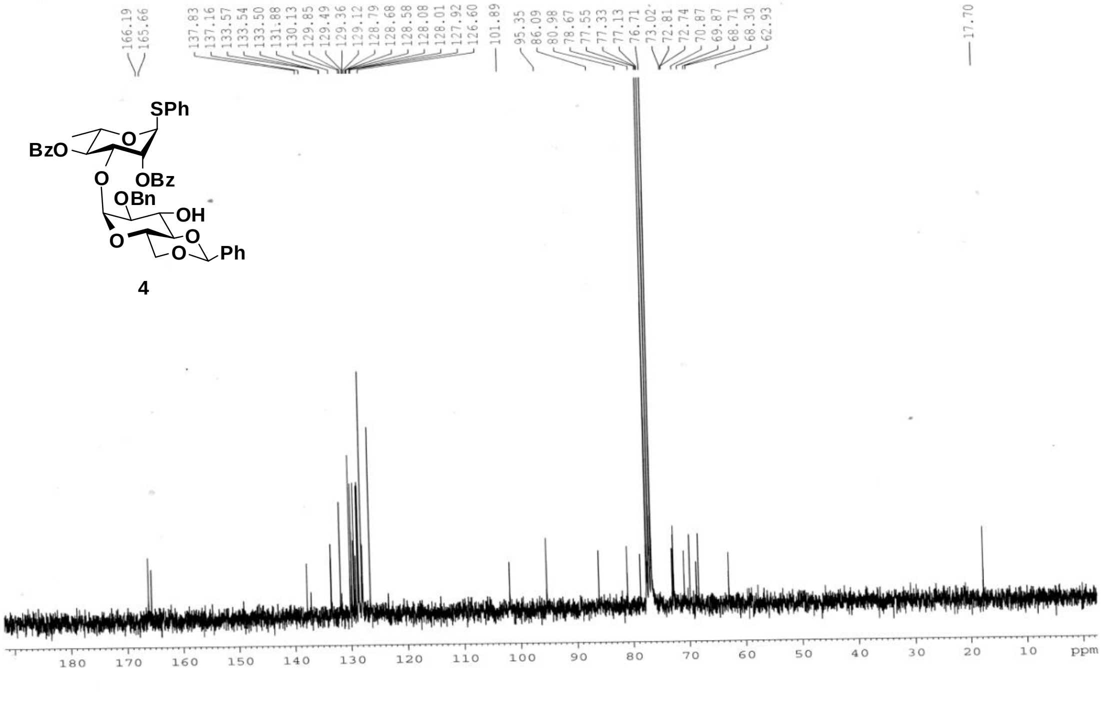

$^1$H-NMR (300 MHz) spectra of compound **21 (CDCl$_3$)**

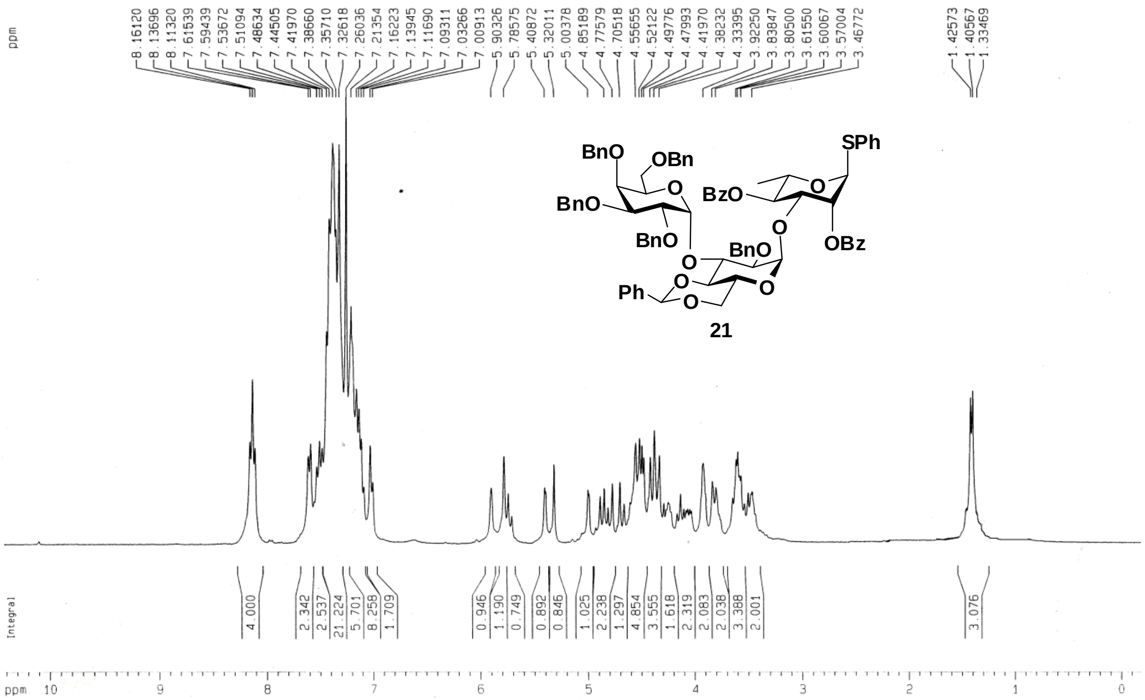

$^{13}$C-NMR (75 MHz) spectra of compound **21 (CDCl$_3$)**

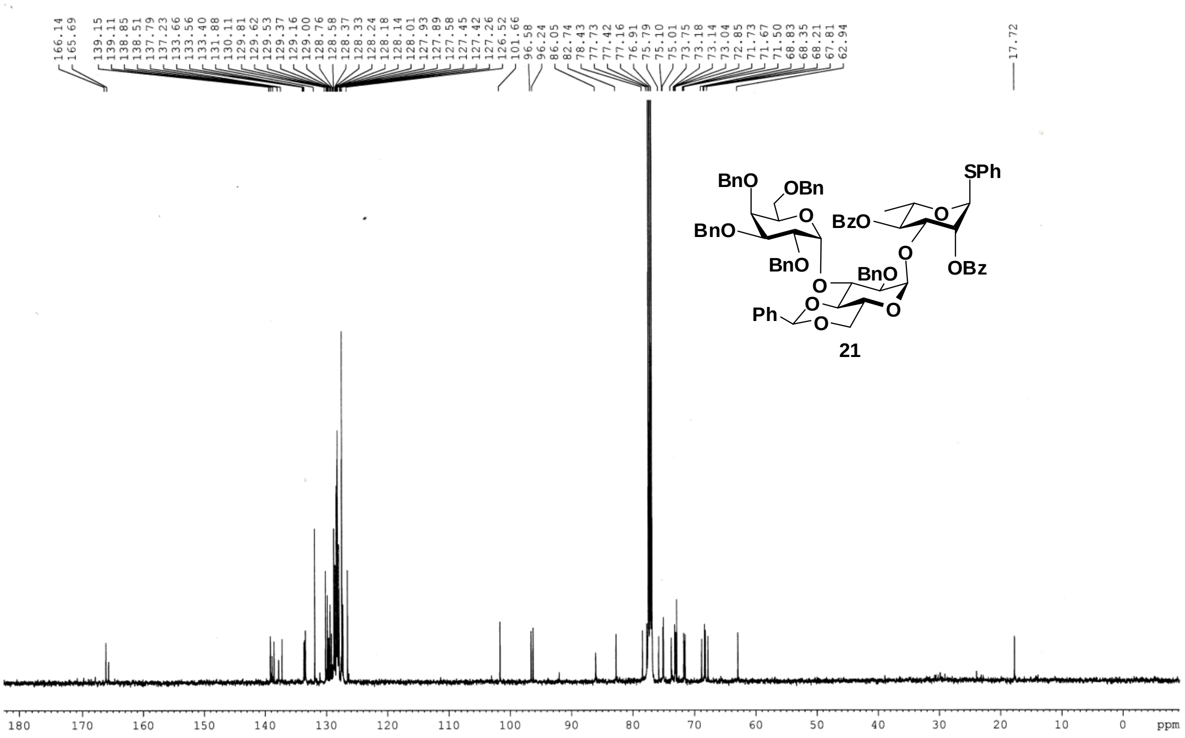

$^{1}$H-NMR (300 MHz) spectra of compound **1 ($CDCl_3$)**

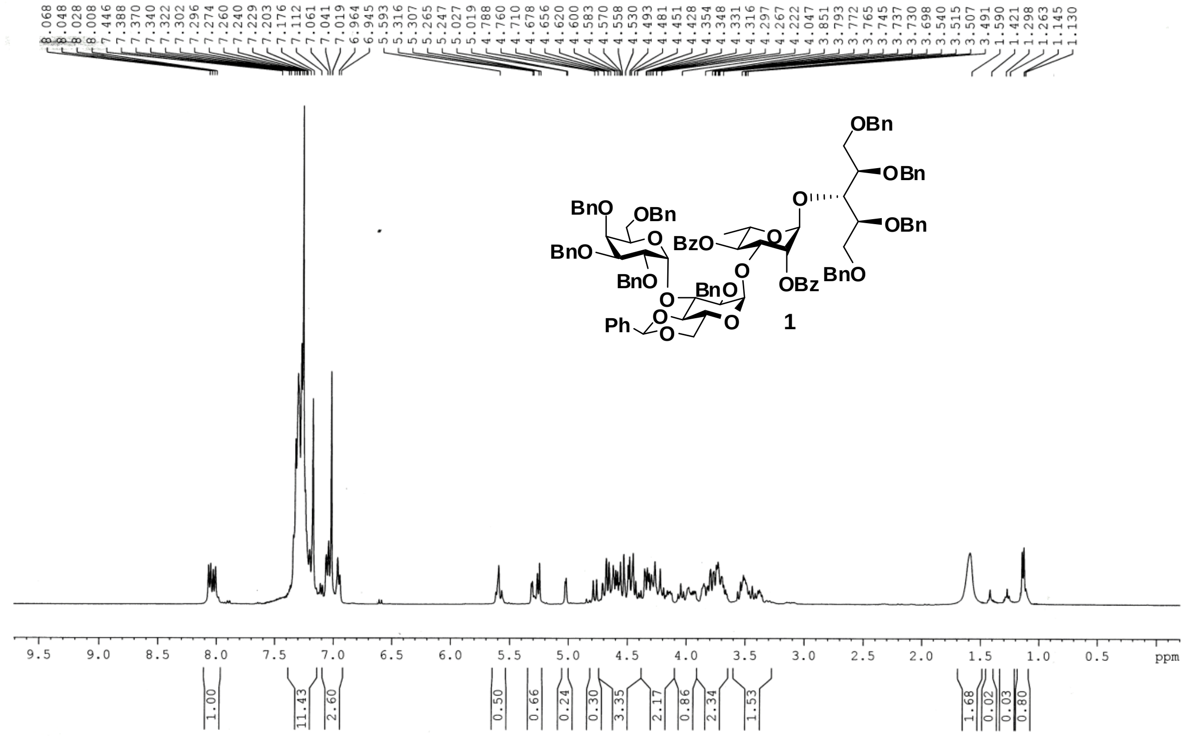


$^{13}$C-NMR (75 MHz) spectra of compound **1 ($CDCl_3$)**

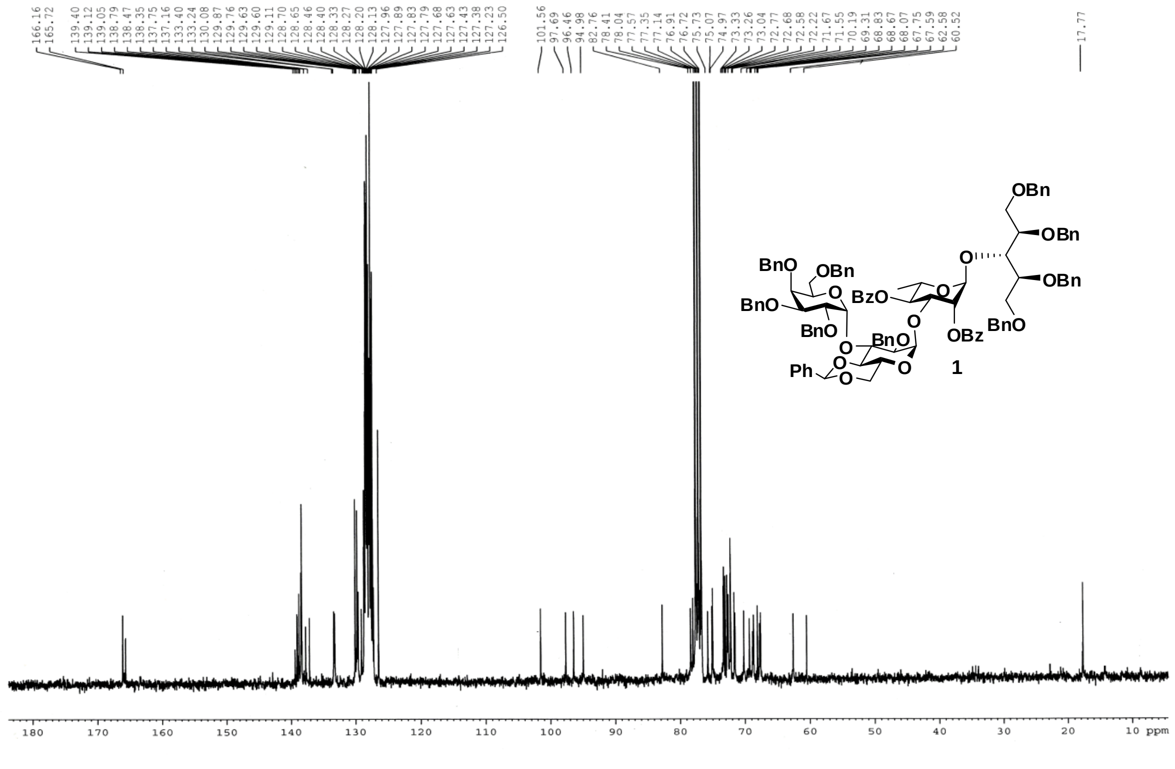

$^{1}$H-NMR (500 MHz) spectra of compound **23 (D$_2$O)**

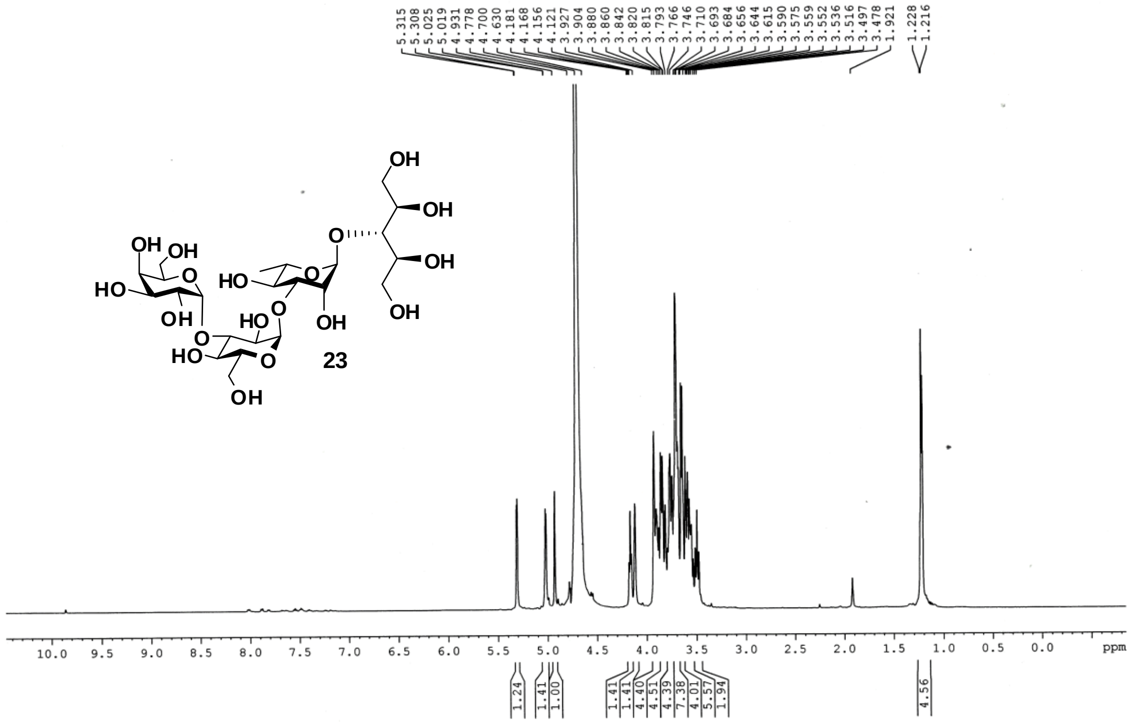


$^{13}$C-NMR (125 MHz) spectra of compound **23 (D$_2$O)**

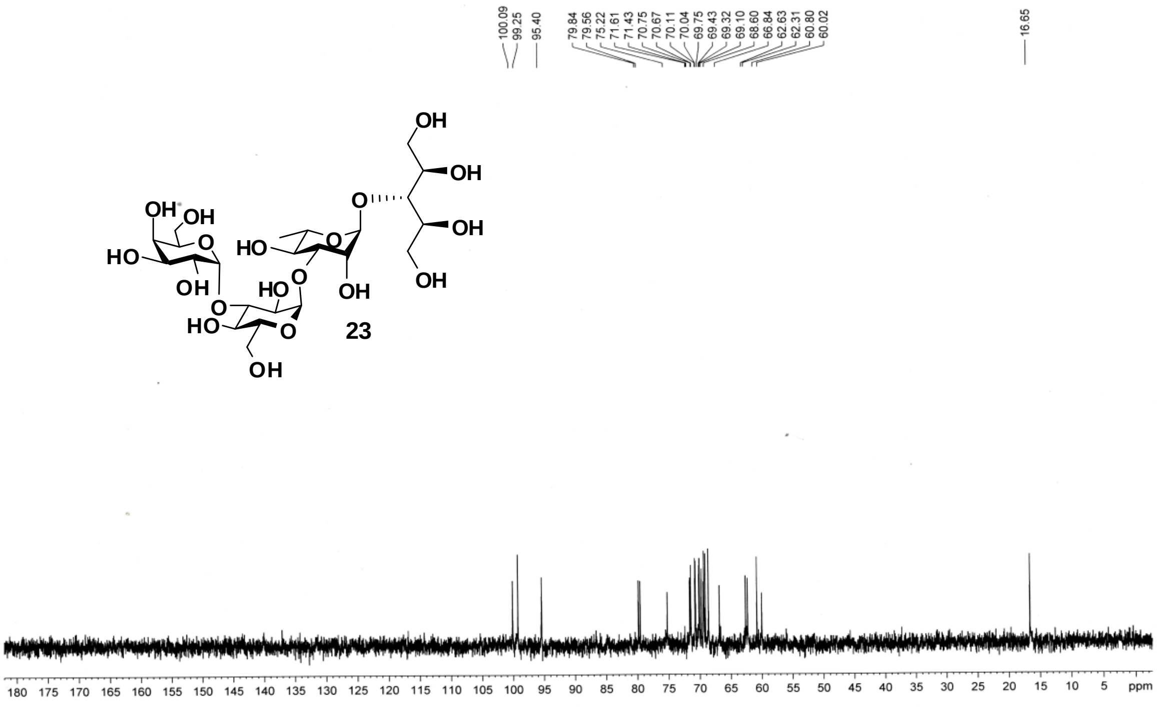

## $^{1}H$-$^{13}C$ Coupled NMR of compound **23**

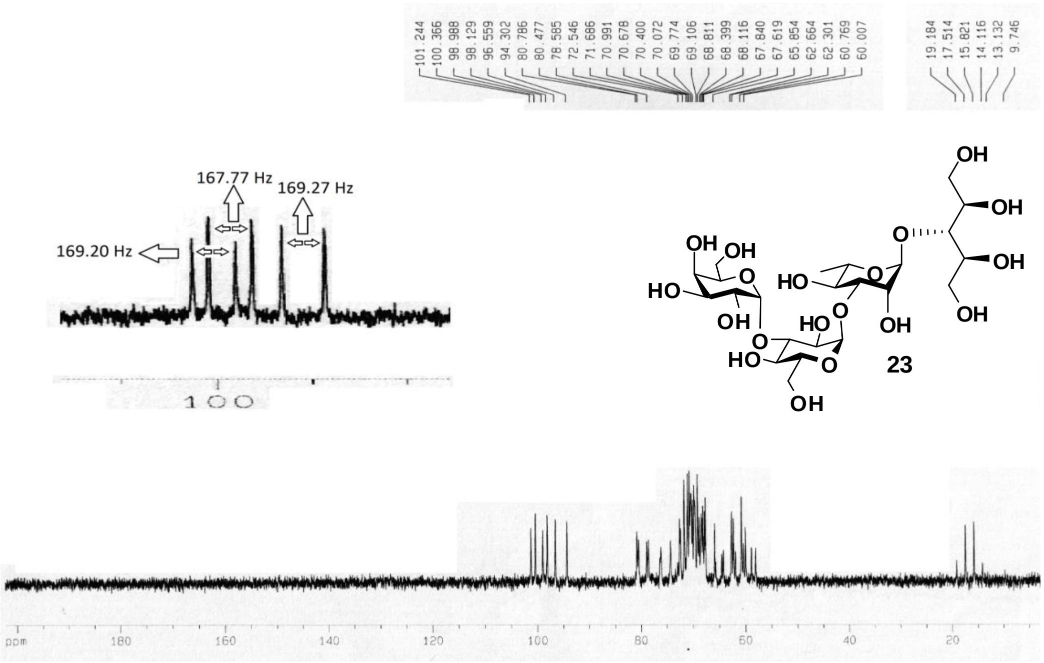


## $^{1}H$-$^{1}H$ COSY NMR spectra of compound **23**

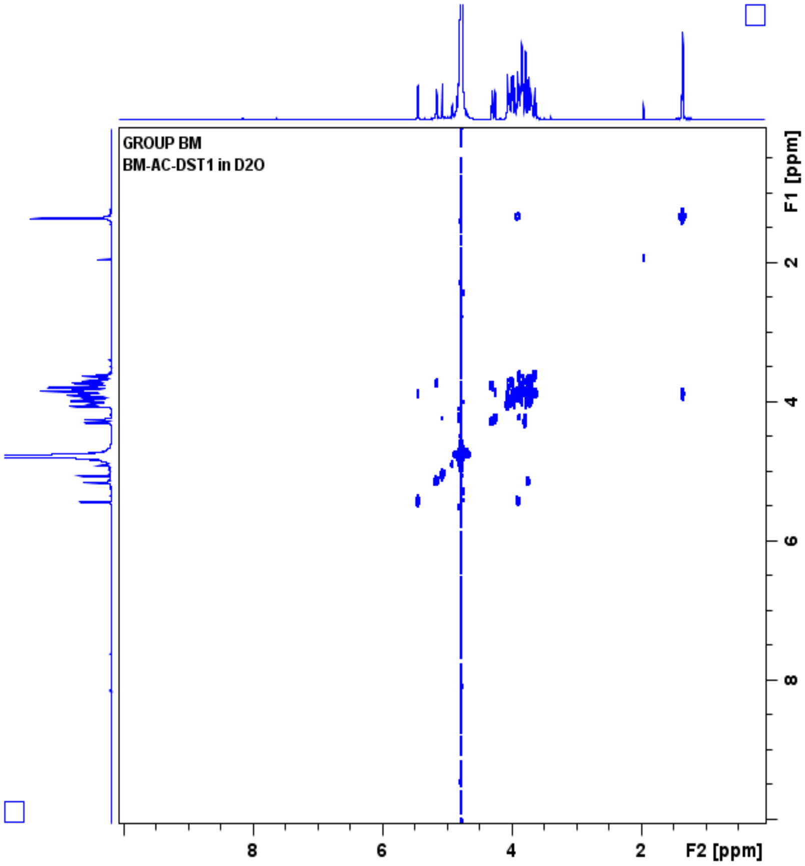

$^1H$-$^1H$ COSY NMR expansion **δ 3.3-5.5.**

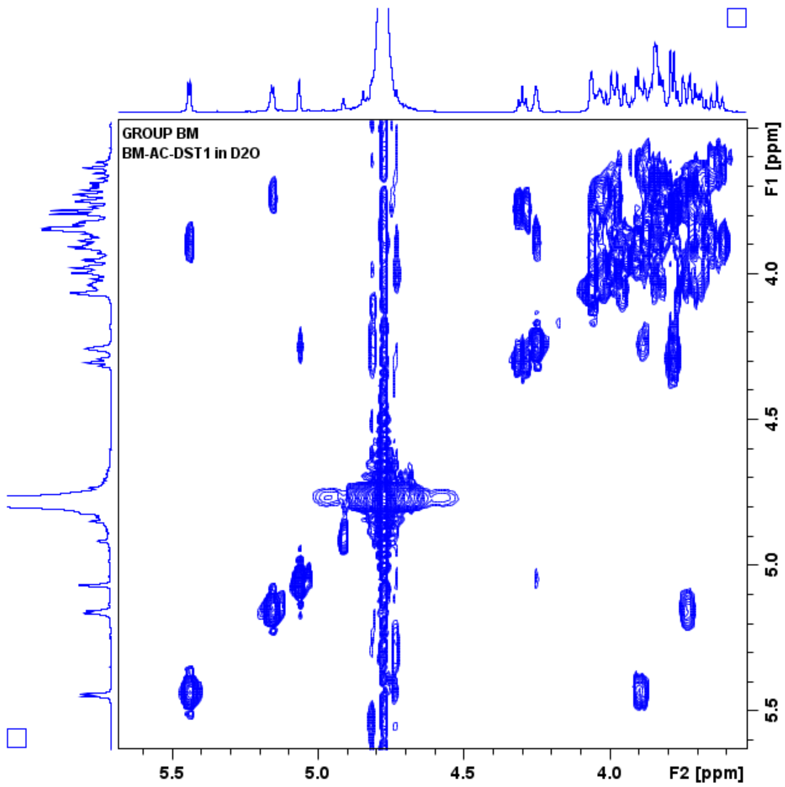


$^1H$-$^1H$ COSY NMR expansion **δ 3.4-4.4.**

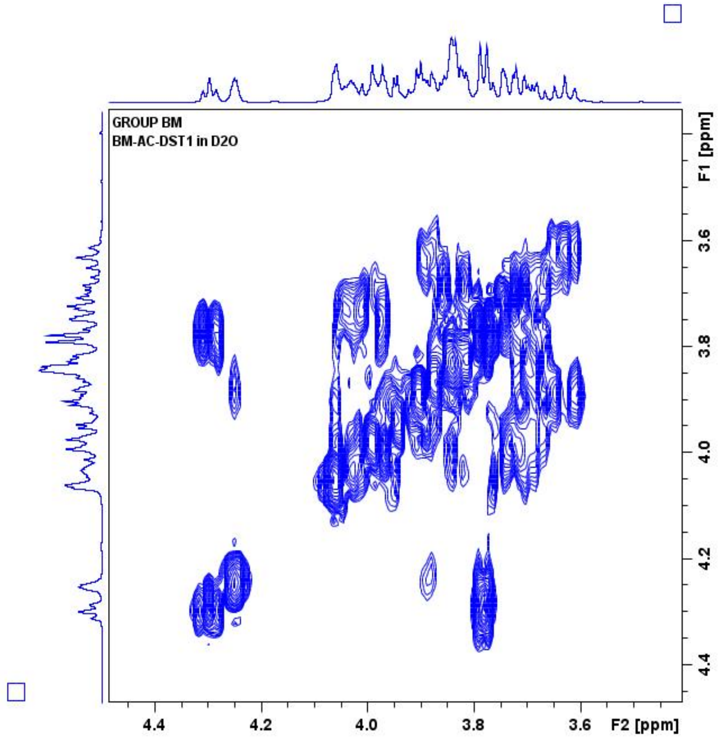

HSQC spectra of compound **23**

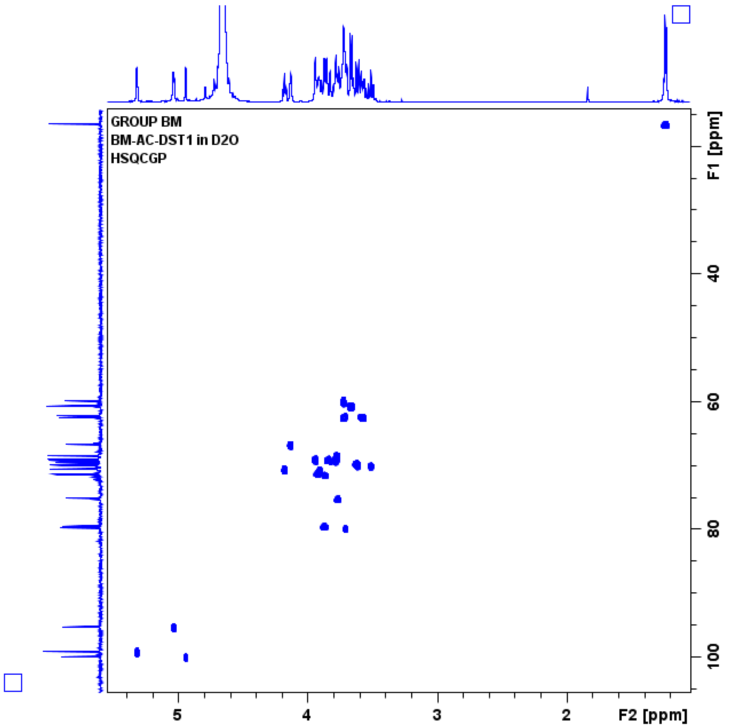


HSQC spectra of compound **23** expansion δ 3.5-4.0 and 58-82

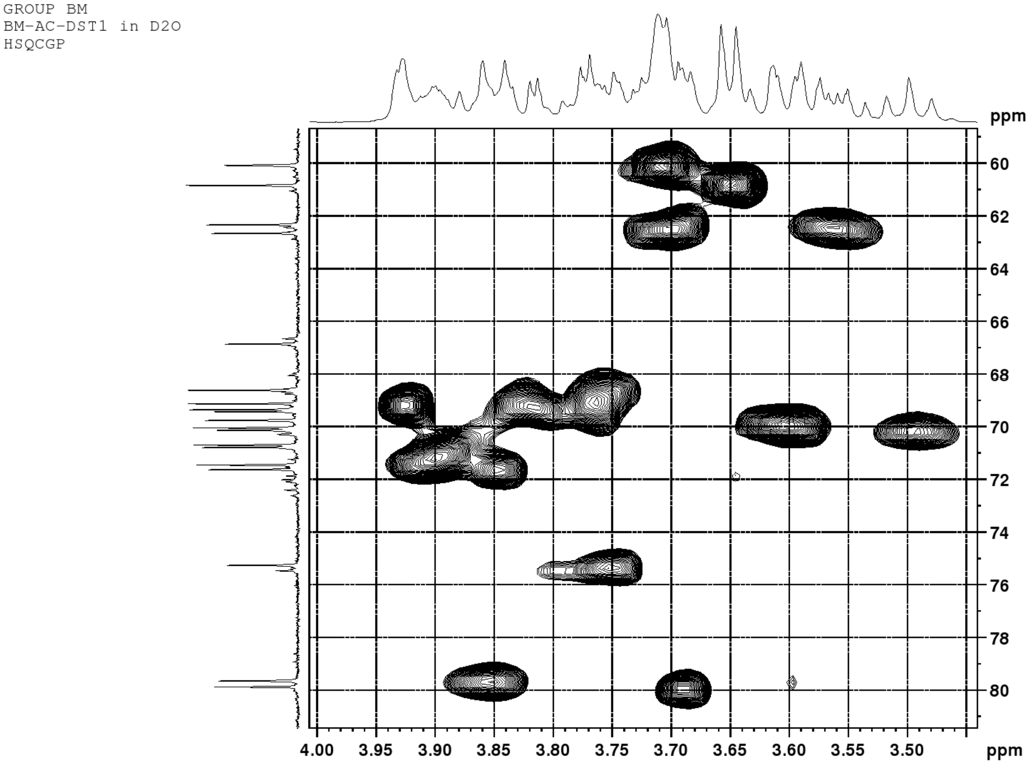

HMBC spectra of compound **23**

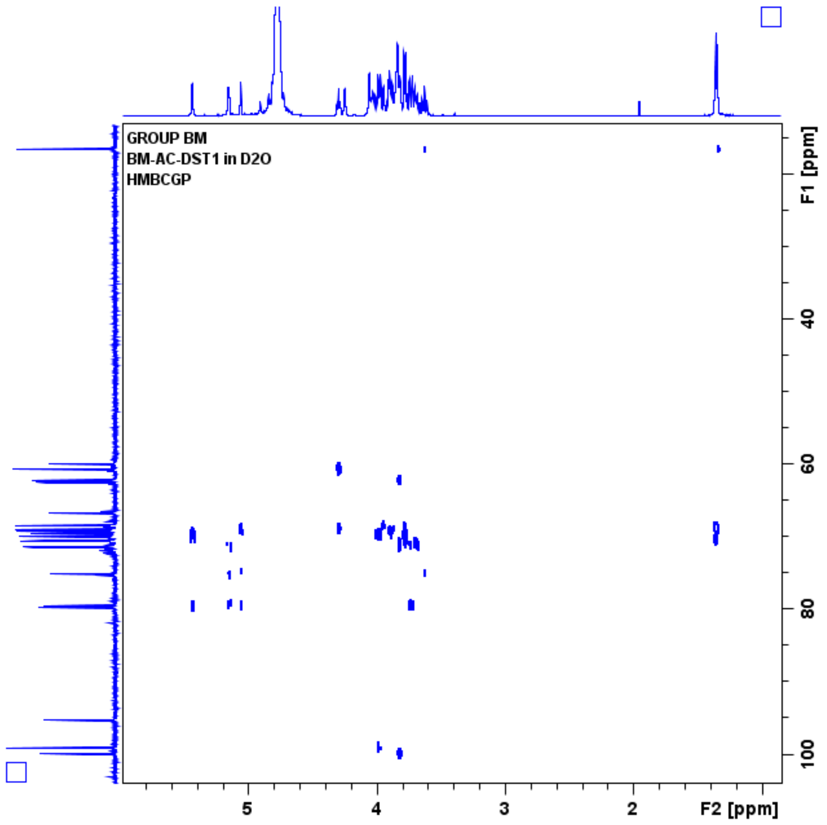


HMBC spectra of compound **23** δ 3.5-5.5 and 60-100

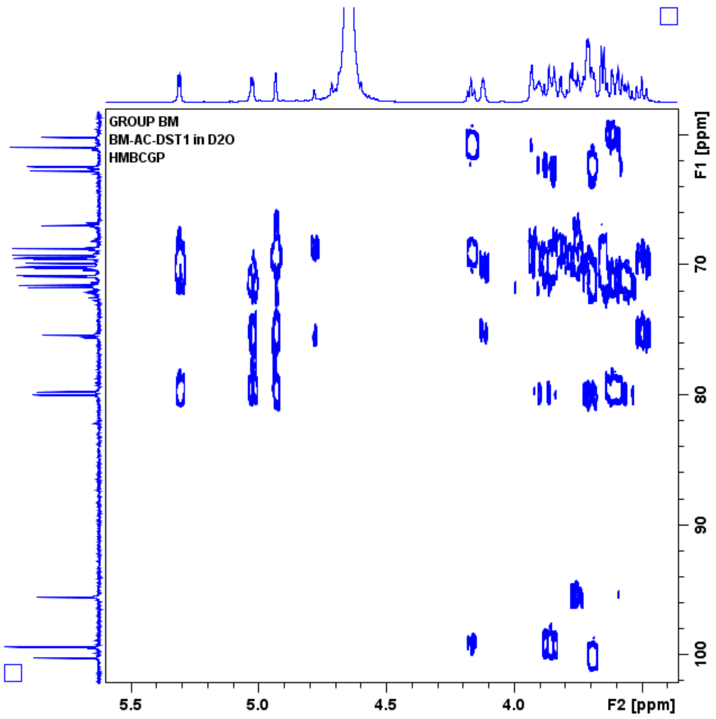

Supplement: File 2 — 1H and 13C NMR of compounds 1, 3a, 4, 5, 6a, 6b, 7, 12a, 19, 20, 21, and 23 and 2D NMR (COSY, HSQC and HMBC) of compound 23. [file Beilstein_J_Org_Chem-14-1095-s002.pdf]
